# The use of an ‘acclimatisation’ heatwave measure to compare temperature-related demand for emergency services in Australia, Botswana, Netherlands, Pakistan, and USA

**DOI:** 10.1371/journal.pone.0214242

**Published:** 2019-03-28

**Authors:** Naomi van der Linden, Thomas Longden, John R. Richards, Munawar Khursheed, Wilhelmina M. T. Goddijn, Michiel J. van Veelen, Uzma Rahim Khan, M. Christien van der Linden

**Affiliations:** 1 Centre for Health Economics Research and Evaluation, University of Technology Sydney, Sydney, Australia; 2 Emergency Department, UC Davis Medical Center, Sacramento, California, United States of America; 3 Emergency Department, Aga Khan University Hospital, Karachi, Pakistan; 4 Emergency Department, Academic Medical Centre, Amsterdam, Netherlands; 5 Princess Marina Hospital & Department of Emergency Medicine, University of Botswana, Faculty of Medicine, Gaborone, Botswana; 6 Emergency Department, Haaglanden Medical Centre, The Hague, Netherlands; Tongji University, CHINA

## Abstract

**Background:**

Heatwaves have been linked to increased risk of mortality and morbidity and are projected to increase in frequency and intensity due to climate change. The current study uses emergency department (ED) data from Australia, Botswana, Netherlands, Pakistan, and the United States of America to evaluate the impact of heatwaves on ED attendances, admissions and mortality.

**Methods:**

Routinely collected time series data were obtained from 18 hospitals. Two separate thresholds (≥4 and ≥7) of the acclimatisation excess heat index (EHIaccl) were used to define “hot days”. Analyses included descriptive statistics, independent samples T-tests to determine differences in case mix between hot days and other days, and threshold regression to determine which temperature thresholds correspond to large increases in ED attendances.

**Findings:**

In all regions, increases in temperature that did not coincide with time to acclimatise resulted in increases in ED attendances, and the EHIaccl performed in a similar manner. During hot days in California and The Netherlands, significantly more children ended up in the ED, while in Pakistan more elderly people attended. Hot days were associated with more patient admissions in the ages 5–11 in California, 65–74 in Karachi, and 75–84 in The Hague. During hot days in The Hague, patients with psychiatric symptoms were more likely to die. The current study did not identify a threshold temperature associated with particularly large increases in ED demand.

**Interpretation:**

The association between heat and ED demand differs between regions. A limitation of the current study is that it does not consider delayed effects or influences of other environmental factors. Given the association between heat and ED use, hospitals and governmental authorities should recognise the demands that heat can place on local health care systems. These demands differ substantially between regions, with Pakistan being the most heavily affected within our study sample.

## Introduction

Heatwaves have been linked to increased risk of mortality and morbidity, and are projected to increase in frequency and intensity due to climate change [1–4]. The health effects of heatwaves include direct heat-related medical conditions (e.g. heat rash, heat oedema, heat syncope, heat cramps, heat exhaustion, and heatstroke), worsening of existing medical conditions (e.g. chronic pulmonary diseases, cardiac conditions, kidney disorders, and psychiatric illness), and problems with medication [5]. Therefore, heatwaves increase the demand for healthcare services in numerous ways.

Increased use of healthcare services during periods of extreme heat is a concern to healthcare professionals and policymakers worldwide. This is evident in the implementation of heatwave risk management plans in a range of countries, including Australia, Germany, Italy, Netherlands, Spain, the United Kingdom and the United States of America (USA) [6]. The severity and impact of heatwaves differs across the globe due to differences in meteorological, socioeconomic (e.g. affordability of air conditioners) and other circumstances, such as the ability to provide rapid and appropriate healthcare to patients in need. The current study uses hospital data from five different countries (Australia, Botswana, Netherlands, Pakistan, and the USA) on five different continents to evaluate the impact of heatwaves.

This study aims to assess to what extent hot days result in increases in emergency department (ED) attendances (for all regions), hospital admissions, and mortality (for a subset of regions: Netherlands, Pakistan and the USA). For the subset of regions, it also evaluates to what extent the case mix (patient age, gender, acuity, and particular symptoms) differs between hot days versus other days. Furthermore, this study evaluates whether different temperature thresholds correspond to large increases in demand for ED services across disparate regions and climates. Climates in the selected regions include a temperate, oceanic climate in both Dutch cities, a hot semi-arid climate in Gaborone, a hot desert climate in Karachi, and a hot-summer Mediterranean climate in Sacramento and Perth.

While prior publications addressed the impact of temperature on mortality [7, 8], few studies related temperature to morbidity as well as mortality [9]. Åström et al. reviewed studies about the impact of heatwaves and elevated temperature on the elderly. They found that studies consistently reported increases in cardiovascular and respiratory mortality, however, the number of studies on morbidity was small [10]. Some studies included a range of relevant outcome measures, such as ambulance call-outs, ED visits and mortality [11]. Studies that did address morbidity more often focused on the association between heat and hospital admissions rather than ED visits, and thus may capture only the most severe non-fatal outcomes [9]. This study overcomes these limitations by including ED attendances, as well as hospital admissions and mortality as outcome measures. It also looks at patients’ presenting complaints, to investigate which type of problems are overrepresented on hot days.

In addition to more evidence on the association between ambient temperature, morbidity and mortality, prior publications have emphasised the need for further research to determine appropriate measures of exposure, to perform more multicity studies with consistent methodology to make it easy to compare and interpret the temperature effects on morbidity across cities, and to investigate threshold temperatures in specific locations [12]. The current study aims to address each of the above, by using a heatwave measure which accounts for acclimatisation, performing the same analyses using data from different regions, and performing threshold analyses.

Existing heatwave definitions vary by temperature metrics, thresholds, and duration, and no consensus exists on which best predicts morbidity and mortality. One of the reasons for this is that thresholds of concern may be different in milder climate regions or early in warm seasons due to regional or temporal acclimatisation [13]. Many heatwave risk management plans do not account for region specific temperature thresholds and acclimatisation. The findings from this study may inform improved design of region-specific heatwave risk management plans and may help healthcare providers to take appropriate action when it is going to be dangerously hot outside.

## Methods

### Data

Routinely collected time series data were obtained from a convenience sample of 18 hospitals in five countries. The data included information on the number of patient visits and mortality per day for each of the hospitals. These data were used to inform the descriptive analyses and threshold regression. For four of the hospitals (in three countries; Netherlands, Pakistan and USA) additional data was available including three or more of the following: patient age, gender, acuity, symptoms, and whether the patient needed to be admitted to the hospital. These data were used to provide a comparison between hot days and other days in terms of patient and visit characteristics, for each of the available variables (available variables differed between hospitals). The available data per site is specified in the “Results—Descriptives” section of this manuscript and concerns the years 2009–2016, or parts of this period. Temperature data were collected through the weather stations closest to the respective hospitals and included the daily maximum temperature and the daily minimum temperature.

Since it was not possible to study the incidence of each type of symptom, health conditions were selected based on published literature. In 49 EDs in France, the health conditions that were seen significantly more frequently during hot periods were: dehydration, hyperthermia, malaise, hyponatremia, renal colic, and renal failure [14]. Based on a study in 16 climate zones throughout California, higher temperatures result in significantly more admissions for acute renal failure, appendicitis, dehydration, ischemic stroke, mental health, non-infectious enteritis, and primary diabetes [13]. In the 2006 California heatwave, data from six geographic regions of California showed significant increases for acute renal failure, cardiovascular diseases, diabetes, electrolyte imbalance, and nephritis [15]. In Taipei, higher temperatures were found to be associated with an increased risk of ED visits for chronic renal failure, diabetes, and accidents [16].

Based on the above associations and data availability, the following symptoms were selected to evaluate their incidence on hot days versus other days: cardiac symptoms, dehydration, diabetes mellitus, heat exhaustion, malaise, psychiatric symptoms, renal/urinary symptoms, respiratory symptoms and stroke.

Many studies, especially those conducted in cooler climates, restricted their analyses to a “warm” season. However, similar to a prior study in North Carolina [9], we decided to include the full calendar years in our analyses since there were heat events throughout the year.

Ethics approval was obtained from the UTS Human Research Ethics Committee, UTS HREC REF NO. 2015000135, and confirmed by the METC Zuidwest Holland, the AMC wetenschapscommissie SEH, the UC Davis Institutional Review Board, the Aga Khan University Ethics Review Committee and the Office of Research and Development of the University of Botswana.

### Heatwave measure

Definitions of heatwaves or “hot days” differ widely. Initially, the exposure measures used in this study were the ones that were outlined in Scalley et al. [17], see Table 1 below. Where a reference value was required, this was set at the 90th percentile daily maximum temperature recorded in the defined period at the respective weather stations. After performing initial, exploratory analyses and to simplify our results, the acclimatisation excess heat index (EHI_A) was chosen as main exposure measure to report on in this paper. The EHI_A captures the impact of a period of notably warmer weather compared to the previous 30 days. Hereby it accounts for temperature as well as time to acclimatise.

**Table 1:** Heatwave measures.

Heatwave measure/ temperature variable	Description	Formulation
Three daily maximum temperature (3DMT)	This is compared to a climate reference value, 3*DMT*_*it*,*k*_	3*DMT*_*it*_ = min(*MaxT*_*it*_, *MaxT*_*it*−1_, *MaxT*_*it*−2_)
Daily average temperature (DAT)	This calculation is used in the following measures (listed below).	*DAT*_*it*_ = (*MaxT*_*it*_ + *MinT*_*it*_) / 2
Three daily average temperature (3DAT)	This is compared to a climate reference value, *MaxT*_*it*,*k*_	3*DAT*_*it*_ = (*DAT*_*it*_ + *DAT*_*it*−1_ + *DAT*_*it*−2_) / 3
Significant excess heat index (EHI_S)	This measure captures the excess heat that coincides with a high daytime temperature that is not dissipated overnight due to an unusually high overnight temperature. This includes the climate reference value in the index’s formulation, *MaxT*_*it*,*k*_	*EHI*_*S*_*it*_ = 3*DAT*_*it*_ − *MaxT*_*it*,*k*_
Acclimatisation excess heat index (EHI_A)	This measure captures the heat stress that is related to a period of warmer weather that is notable in comparison to the previous 30 days. It captures a short-term (acclimatisation) temperature anomaly.	*EHI*_*A*_*it*_ = 3*DAT*_*it*_ − (*DAT*_*it*−1_ + ⋯ + *DAT*_*it*−30_) / 30
Excess Heat Factor (EHF)	This measure combines two measures to simultaneously capture the effect of Excess Heat (EHI_S) and Heat Stress (EHI_A). Heatwave conditions exist when the EHF is positive.	*EHF*_*it*_ = *EHF*_*S*_*it*_ × max(1, *EHI*_*A*_*it*_)

Note: this Table is based on Table 1 from Scalley, Spicer (17).

**Fig 1:**
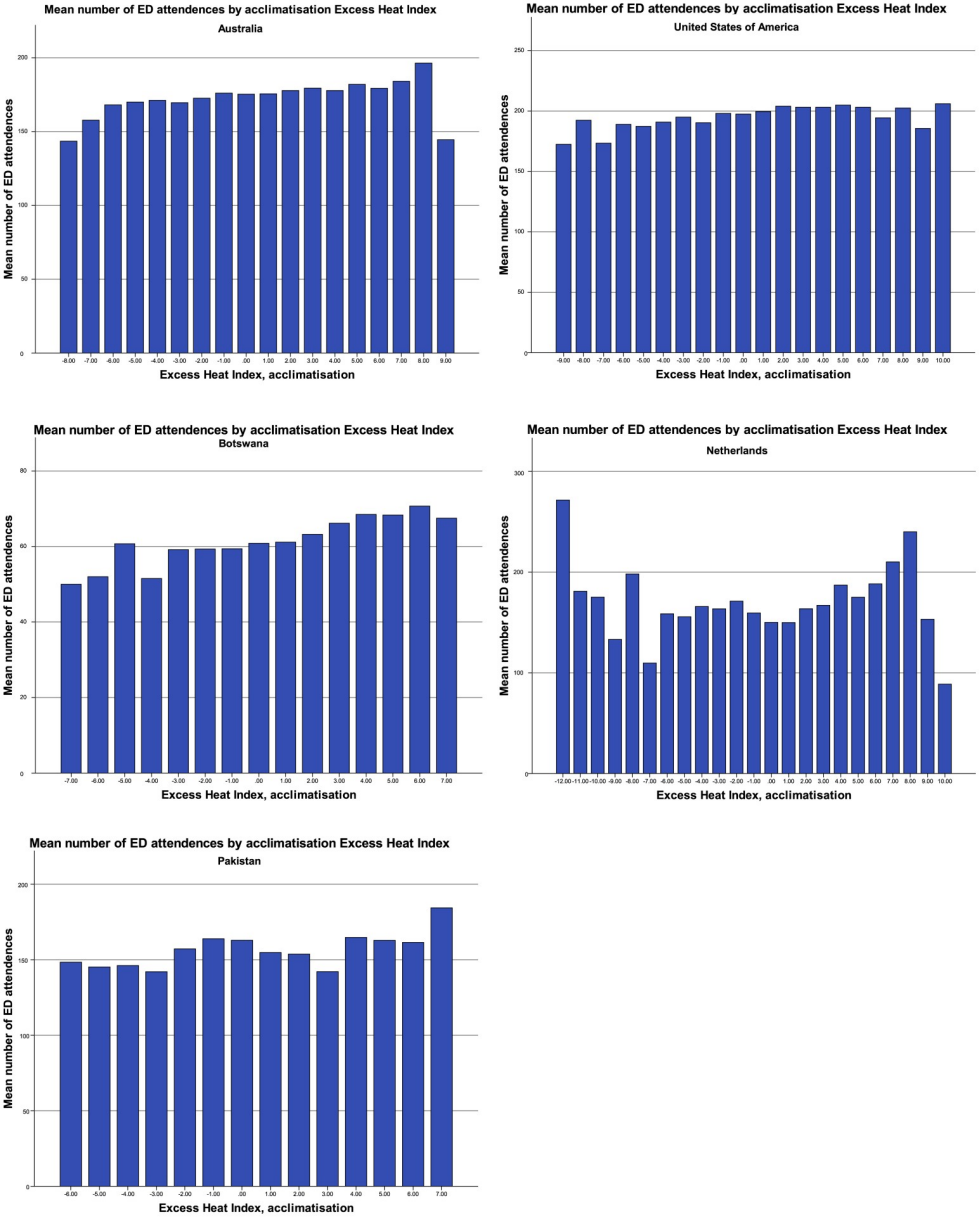
Mean number of ED attendances dependent on the acclimatisation excess heat index.

Two separate thresholds were used to define “hot days”: (1) days with an EHI_A ≥ 4, and (2) days with an EHI_A ≥ 7, based on use in a prior study [7]. It was decided to use these same thresholds after eyeballing Fig 1 (see Results section), which did not identify alternative EHI_A thresholds associated with clear increases in the mean number of ED attendances in the selected regions. This is in line with the results of the threshold regression (see “Threshold regression” in the Results section).

When a day has an EHI_A of four (or seven), the average temperature in the last three days is four (or seven) degrees Celsius higher than the average temperature in the last thirty days. Therefore, the EHI_A captures the impact of a period of notably warmer weather compared to the previous thirty days (accounting for temperature as well as time to acclimatize).

### Analyses

Analyses included descriptive statistics to provide data characteristics, independent samples T-tests to determine differences in case mix between hot days and other days, and threshold regression to determine which temperature thresholds correspond to large increases in demand for ED services.

The time-series threshold regression was performed using the EHI_A as the heat-related measure. This regression approach has been used to investigate whether there are thresholds that are relevant to the number of daily ED services. Thresholds have been found for temperature-related mortality using a similar approach in [7] and this analysis focuses on whether the same is true for ED services. These regressions were performed in Stata using the ‘threshold’ command with the data specified as time-series data for each hospital. Explanatory variables for the regression model included day of the week, month, and year. These variables control for the seasonal and annual changes in ED demand so that the estimates associated with ambient temperature do not include these factors. The model allowed for multiple thresholds and was specified as:

$$EDAtt_{it}=\{\begin{array}{c} T_{t}\beta_{1}+Z_{t}\alpha +e_{t},T_{t}<\gamma_{1}\\ T_{t}\beta_{2}+Z_{t}\alpha +e_{t},\gamma_{1}\leq T_{t}<\gamma_{2}\\ T_{t}\beta_{3}+Z_{t}\alpha +e_{t},T_{t}\geq \gamma_{2} \end{array}$$ (1)

where *Z*_*t*_ is a vector of explanatory variables, *T*_*t*_ is the heat-related variable, and EDAtt refers to the number of ED attendances. The number of ED attendances per population size was also considered as a dependent variable, but the size of the appropriate population for these hospitals was unknown. For countries with more than one hospital, the threshold analysis was performed for the hospital with the largest number of daily ED attendances.

## Results

### Descriptives

Table 2 provides characteristics of the data used in this study. The temperature ranges for the regions of Australia, Botswana, Pakistan and the USA are similar, however, the Netherlands had cooler temperatures. The largest ED demand occurred at Fiona Stanley hospital in Perth.

**Table 2:** Available data per site.

Country	City / region	Hospital	Period	Temperature range (°C): min, max	Mean maximum temperature (°C)	Mean number of daily ED attendances
Australia	Perth	Armadale/Kelmscott District Memorial Hospital	01/01/2014-31/12/2016	1.6, 44.0	25.0	166
		Joondalup Health Campus		0.6, 44.4	25.0	268
		King Edward Memorial Hospital for Women		0.6, 44.4	25.0	36
		Princess Margaret Hospital for Children		0.6, 44.4	25.0	181
		Rockingham General Hospital		0.4, 44.4	23.9	146
		Royal Perth Hospital		0.6, 44.4	25.0	207
		Sir Charles Gairdner Hospital		3.4, 44.3	24.4	190
		Fremantle Hospital	01/01/2014-03/02/2015	0.1, 43.7	25.7	160
		Fiona Stanley Hospital	03/02/2015-31/12/2016	0.2, 43.3	24.2	278
		St. John of God Midland Hospital	24/11/2015-31/12/2016	0.9, 43.0	24.9	169
		Swan District Hospital	01/01/2014-24/11/2015	0.1, 44.2	25.6	132
Botswana	Gaborone	Princess Marina Hospital	25/08/2015-31/12/2016	-2.4, 42.8	30.2	62
Netherlands	Amsterdam	Academic Medical Centre	05/07/2010-31/12/2016	-18.8, 33.7	14.5	86
	Den Haag	HMC Antoniushove	01/01/2009-31/12/2016	-11.5, 36.5	14.2	54
	Den Haag	HMC Bronovo	13/08/2012-31/12/2016	-9.0, 36.5	14.5	53
	Den Haag	HMC Westeinde	01/01/2009-31/12/2016	-11.5, 36.5	14.2	139
Pakistan	Karachi	Aga Khan University Hospital	01/01/2009-14/10/2016	6.0, 44.8	32.8	157
USA	Sacramento	UC Davis Medical Center	01/01/2009-31/12/2016	-6.0, 42.0	23.9	197

Fig 1 provides the mean number of ED attendances by EHI_A. Similar histograms using other exposure measures are provided as online Supplementary Material (S2 Fig). In each region, the number of ED visits increases with higher EHI_A. In some of the countries, the highest EHI_A is associated with a slightly lower number of ED visits than the penultimate classification of EHI_A. Potentially this is due to a delayed effect of heat on ED demand, or a tendency to stay inside with extremely hot temperatures. In the Netherlands, ED demand increases with high EHI_A but also with extremely low EHI_A values. This reflects the negative degree days that the other regions where not subject to. In all settings, the EHI_A indicator had an average that is close to zero and closely coincides with a normal distribution, suggesting it performs similarly across different climates (see S1 Fig in the Supplementary Material). An outlier is the ED demand in the Netherlands as it has larger negative values of the EHI_A measure.

### Hot days versus other days

Table 3 provides the number of ED visits and the proportion of patients by patient/visit characteristics on hot days versus other days.

**Table 3:** Emergency department visits and patient/visit characteristics on hot days versus other days.

	Hot days, EHIaccl ≥ 4	Other days, EHIaccl < 4	Sig^1^	Hot days, EHIaccl ≥ 7	Other days, EHIaccl < 7	Sig^1^
**Netherlands, Amsterdam**						
**N**	186	2,185		36	2,335	
**Age, mean % (sd)**						
<5	9.1 (3.0)	8.7 (3.5)	0.147	9.2 (2.9)	8.7 (3.5)	0.362
5–11	6.8 (3.0)	6.1 (2.8)	0.000*	7.5 (3.4)	6.1 (2.8)	0.005*
12–17	6.5 (2.8)	6.7 (2.9)	0.614	6.0 (2.5)	6.7 (2.9)	0.200
18–34	24.0 (4.9)	24.5 (5.1)	0.238	24.1 (5.3)	24.5 (5.1)	0.646
35–64	37.3 (5.4)	37.5 (5.7)	0.617	37.0 (5.7)	37.5 (5.7)	0.591
65–74	8.6 (3.2)	9.1 (3.5)	0.110	8.9 (3.0)	9.0 (3.5)	0.781
75–84	5.4 (2.3)	5.4 (2.6)	0.845	5.1 (2.6)	5.4 (2.5)	0.463
85+	2.6 (1.5)	2.7 (1.5)	0.509	3.0 (1.6)	2.7 (1.5)	0.424
**Proportion of males, mean (sd)**	53.0 (5.5)	53.2 (5.8)	0.654	52.9 (4.9)	53.2 (5.8)	0.772
**Acuity, mean % (sd)**						
Non-urgent (blue, green)	32.0 (18.7)	31.1 (18.1)	0.486	28.3 (19.3)	31.2 (18.2)	0.347
Urgent (yellow, orange, red)	35.1 (21.5)	38.2 (22.5)	0.072	33.8 (24.1)	38.0 (22.4)	0.261
Not triaged^2^	32.8 (35.3)	30.7 (35.9)	0.445	37.8 (37.5)	30.8 (35.9)	0.245
**Proportion of patients admitted, mean % (sd)**^3^	19.6 (7.1)	20.3 (7.3)	0.472	15.5 (6.9)	20.3 (7.3)	0.040*
**Proportion of patients died, mean % (sd)**^3^	0.1 (0.3)	0.1 (0.4)	0.455	0.1 (0.4)	0.1 (0.4)	0.950
**Netherlands, The Hague (3 locations)**						
**N**	193	2,729		22	2,900	
**Age, mean % (sd)**						
<5	5.2 (1.7)	5.2 (1.7)	0.798	5.9 (2.3)	5.2 (1.7)	0.044*
5–11	6.2 (1.9)	5.5 (1.9)	0.000*	6.1 (1.8)	5.6 (1.9)	0.232
12–17	6.8 (2.0)	6.7 (2.2)	0.476	7.1 (1.5)	6.7 (2.2)	0.363
18–34	26.1 (4.4)	25.7 (4.3)	0.211	24.4 (4.9)	25.7 (4.3)	0.139
35–64	35.0 (3.4)	35.6 (3.5)	0.036*	34.0 (3.5)	35.6 (3.5)	0.041*
65–74	9.0 (2.3)	9.2 (2.4)	0.368	9.7 (2.3)	9.2 (2.4)	0.342
75–84	7.5 (2.1)	7.8 (2.2)	0.069	8.5 (2.4)	7.8 (2.2)	0.113
85+	4.1 (1.6)	4.3 (1.7)	0.091	4.2 (2.1)	4.3 (1.7)	0.939
**Proportion of males, mean (sd)**	51.2 (3.4)	50.9 (3.5)	0.269	51.2 (3.2)	50.9 (3.5)	0.723
**Acuity, mean % (sd)**						
Non-urgent (blue, green)	46.0 (8.3)	43.6 (7.8)	0.000*	43.0 (8.2)	43.8 (7.8)	0.661
Urgent (yellow, orange, red)	43.0 (7.2)	44.8 (8.3)	0.004*	44.7 (10.4)	44.7 (8.2)	0.992
Not triaged	11.0 (9.9)	11.6 (10.0)	0.445	12.3 (11.0)	11.6 (10.0)	0.724
**Proportion of patients admitted, mean % (sd)**	17.5 (6.2)	17.5 (7.7)	0.871	19.9 (4.2)	17.4 (7.6)	0.132
**Proportion of patients died, mean % (sd)**	0.1 (0.2)	0.1 (0.2)	0.885	0.0 (0.1)	0.1 (0.2)	0.381
**Presenting complaints**						
Cardiac	4.8 (3.6)	6.7 (3.3)	0.000*	6.3 (3.3)	6.5 (3.3)	0.788
Diabetes Mellitus	0.2 (0.3)	0.2 (0.3)	0.021*	0.3 (0.3)	0.2 (0.3)	0.797
Malaise	3.9 (3.4)	5.3 (3.3)	0.000*	4.6 (2.9)	5.2 (3.3)	0.515
Psychiatric	1.2 (1.1)	1.6 (1.1)	0.000*	1.1 (0.9)	1.6 (1.1)	0.069
Renal/urinary	1.0 (0.9)	1.5 (1.0)	0.000*	1.3 (0.8)	1.4 (1.0)	0.517
Respiratory	3.4 (2.7)	4.7 (2.7)	0.000*	5.2 (2.5)	4.6 (2.8)	0.423
**Pakistan**						
**N**	104	2,690		3	2791	
**Age, mean % (sd)**						
<5	18.3 (3.5)	18.2 (4.0)	0.765	15.4 (2.9)	18.2 (4.0)	0.211
5–11	7.6 (2.4)	7.5 (2.5)	0.570	7.1 (1.7)	7.5 (2.5)	0.793
12–17	4.3 (1.5)	4.7 (1.9)	0.080	5.8 (0.9)	4.7 (1.9)	0.308
18–34	21.3 (4.3)	22.5 (4.0)	0.002*	22.2 (2.5)	22.4 (4.0)	0.903
35–64	32.0 (4.3)	32.0 (4.6)	0.992	32.0 (1.0)	32.0 (4.6)	0.988
65–74	9.3 (2.3)	8.9 (2.7)	0.152	10.4 (1.6)	8.9 (2.7)	0.337
75–84	5.8 (2.1)	5.0 (2.0)	0.000*	5.5 (2.7)	5.0 (2.0)	0.668
85+	1.4 (1.1)	1.3 (1.0)	0.226	1.6 (0.8)	1.3 (1.0)	0.591
**Proportion of males, mean (sd)**	52.4 (4.3)	53.2 (4.8)	0.087	52.3 (3.8)	53.1 (4.7)	0.752
**Acuity, mean % (sd)**						
Non-urgent (blue, green)	18.2 (7.1)	18.0 (7.5)	0.721	17.0 (9.0)	18.0 (7.5)	0.818
Urgent (yellow, orange, red)	81.8 (7.1)	82.0 (7.1)	0.721	83.0 (9.0)	82.0 (7.5)	0.818
Not triaged	0	0		0	0	
**Proportion of patients admitted, mean % (sd)**	17.5 (18.1)	22.0 (16.9)	0.008*	14.2 (24.5)	21.9 (17.0)	0.432
**Proportion of patients died, mean % (sd)**	0.7 (1.0)	0.8 (1.1)	0.084	0.6 (1.0)	0.8 (1.1)	0.647
**USA**						
**N**	192	2,730		16	2,906	
**Age, mean % (sd)**						
<5	9.4 (2.5)	9.2 (2.5)	0.232	8.4 (2.5)	9.2 (2.5)	0.223
5–11	5.6 (1.9)	5.2 (1.8)	0.016*	5.0 (1.7)	5.3 (1.8)	0.538
12–17	4.9 (1.6)	4.9 (1.7)	0.991	4.7 (1.8)	4.9 (1.7)	0.721
18–34	26.1 (3.5)	26.0 (3.5)	0.503	28.3 (3.6)	25.9 (3.5)	0.008*
35–64	41.0 (3.9)	41.0 (3.9)	0.984	41.9 (3.9)	41.0 (3.9)	0.367
65–74	6.5 (1.9)	6.8 (2.1)	0.016*	6.2 (2.2)	6.8 (2.1)	0.234
75–84	4.1 (1.5)	4.3 (1.6)	0.055	3.2 (1.3)	4.3 (1.6)	0.008*
85+	2.4 (1.2)	2.5 (1.3)	0.270	2.2 (1.2)	2.5 (1.3)	0.300
**Proportion of males, mean (sd)**	50.3 (3.6)	50.1 (3.8)	0.477	47.7 (3.9)	50.1 (3.8)	0.010*
**Acuity, mean % (sd)**						
Non-urgent (blue, green)	16.6 (4.7)	15.5 (4.2)	0.001*	18.1 (5.4)	15.6 (4.2)	0.020*
Urgent (yellow, orange, red)	82.1 (4.8)	82.9 (5.3)	0.031*	81.0 (5.3)	82.9 (5.2)	0.155
Not triaged	1.3 (1.0)	1.6 (4.1)	0.444	0.9 (0.8)	1.5 (4.0)	0.537
**Proportion of patients admitted, mean % (sd)**	2.3 (1.9)	2.3 (2.2)	0.980	3.0 (3.1)	2.3 (2.2)	0.232
**Proportion of patients died, mean % (sd)**	0.2 (0.3)	0.2 (0.4)	0.213	0.2 (0.3)	0.2 (0.4)	0.730
**Presenting complaints**						
Cardiac	7.2 (1.9)	7.2 (2.1)	0.795	7.6 (1.7)	7.2 (2.1)	0.491
Dehydration	0.1 (0.2)	0.1 (0.2)	0.510	0.1 (0.2)	0.1 (0.2)	0.670
Diabetes Mellitus	0.6 (0.6)	0.5 (0.5)	0.165	0.7 (0.6)	0.5 (0.5)	0.249
Heat exhaustion	0.0 (0.2)	0.0 (0.1)	0.000*	0.0 (0.2)	0.0 (0.1)	0.209
Malaise	0.0 (0.0)	0.0 (0.1)	0.166	0.0 (0.0)	0.0 (0.1)	0.593
Psychiatric	5.1 (2.0)	5.2 (1.8)	0.324	5.3 (2.4)	5.2 (1.8)	0.782
Renal/urinary	0.4 (0.4)	0.4 (0.5)	0.879	0.6 (0.6)	0.4 (0.5)	0.100
Respiratory	1.9 (1.4)	2.2 (1.5)	0.011*	1.4 (1.1)	2.2 (1.5)	0.033*
Stroke	0.7 (0.7)	0.9 (0.7)	0.016*	0.8 (0.6)	0.8 (0.7)	0.649

^1^ 2-tailed, equal variances assumed.

^2^ The high proportion of patients who were not triaged is due to periods of missing data as opposed to continuously low triage rates.

^3^ This data was only available for part of the period (from 01/01/2014 onwards). The number of hot days in this period was 10 (EHIaccl ≥ 7) or 66 (EHIaccl ≥ 4).

* The asterisks indicate statistical significance.

During hot days in Sacramento, California (USA) and in The Netherlands (both Amsterdam and The Hague), significantly more children < 12 years old ended up in the ED. On days with an EHIaccl ≥ 4, each of the three Western hospitals had a significantly higher proportion of children 5–11 years old. In Sacramento, the number of adults 18–34 years old was also higher on extremely hot days (≥ 7) than on other days. Other age categories were underrepresented during hot days, such as patients in the age range of 35–64 years in The Hague, and patients in the age range of 65–74 years (EHIaccl ≥ 4) and 75–84 years (EHIaccl ≥ 7) in Sacramento. In Karachi, the findings were opposite. During hot days, more elderly people (75–84 years old) attended the ED, and less patients 18–34 years old.

In The Hague, hot days (EHIaccl ≥ 4) were associated with more non-urgent visits, and, counterintuitively, with less patients with symptoms in the predefined categories. In Sacramento, the proportion of patients with “heat exhaustion” was higher on hot days (EHIaccl ≥ 4), but numbers were small. Both in Karachi (EHIaccl ≥ 4) and Amsterdam (EHIaccl ≥ 7), the proportion of patients admitted through the ED was smaller on hot days.

Results for admissions and mortality are provided as Supplementary Material (S1 and S2 Tables) but should be interpreted with caution due to small sample sizes. Non-significant findings may therefore represent either the lack of an association or the analysis being underpowered. However, the results do show that hot days were associated with more patient admissions in the age range 5–11 years in Sacramento (EHIaccl ≥ 4), and for age range 65–74 years in Karachi (EHIaccl ≥ 4), and 75–84 years in The Hague (EHIaccl ≥ 7). Furthermore, patients with psychiatric symptoms in The Hague were more likely to die during hot days (EHIaccl ≥ 4).

### Threshold analysis

**Fig 2:**
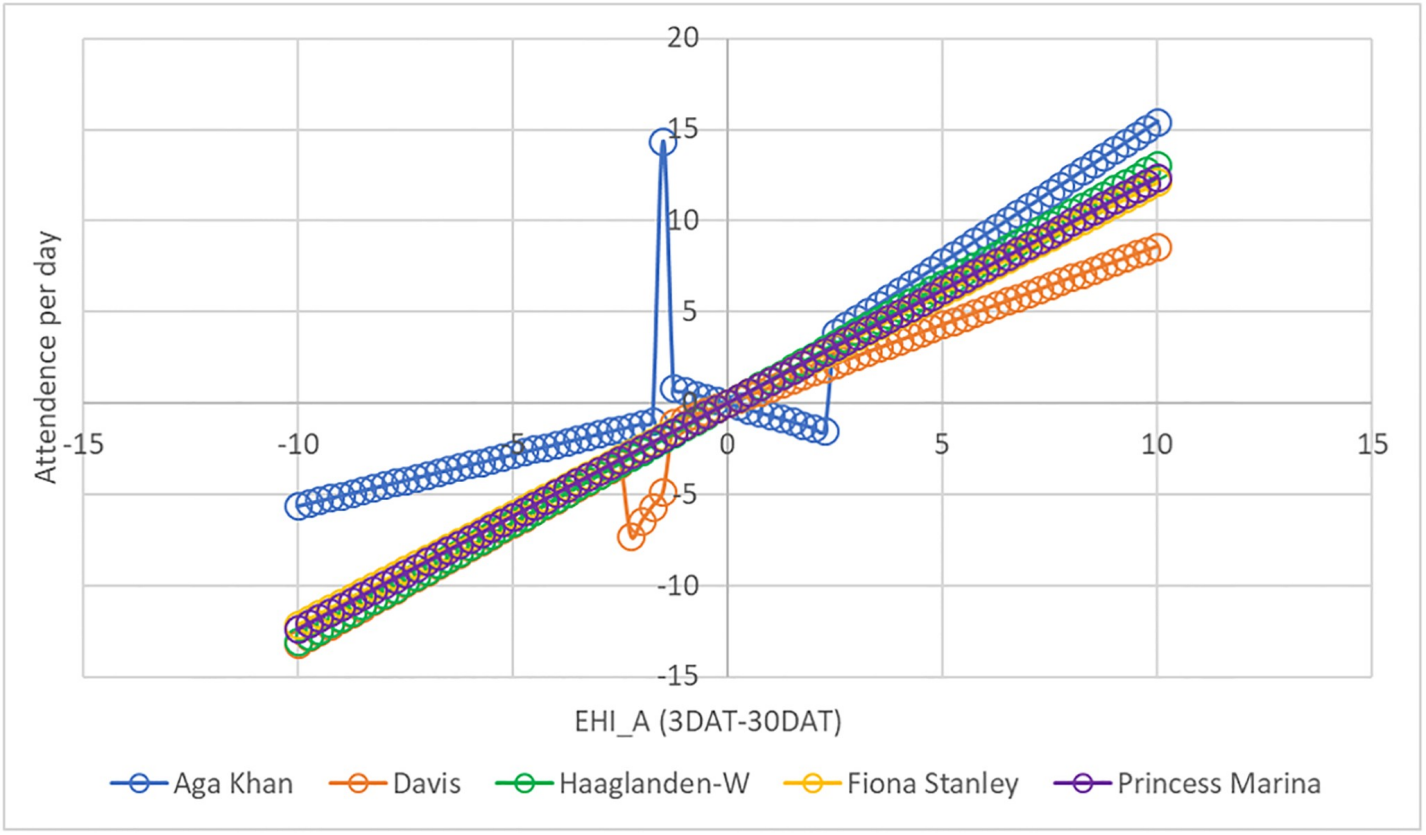
Impact of acclimatisation excess heat index on emergency department attendances, threshold regression.

Fig 2 and Table 4 provide the results of the threshold regression (for the full regression results, see S3 Table). In all settings, increases in temperature that do not coincide with time to acclimatise resulted in increases in ED attendances. This was especially true for the Aga Khan University Hospital, where ED attendances rose rapidly with increasing temperatures. The trend for Aga Khan University Hospital is broken by one outlier, due to a particularly high demand for ED services on one particular day (24 October 2010), which coincided with a Dengue outbreak.

**Table 4:** Estimated thresholds of acclimatisation excess heat index for five hospitals.

Country	Pakistan	USA	Australia	Botswana	Netherlands
**Hospital**	Aga Khan University Hospital, Karachi	UC Davis Medical Center, Sacramento	Fiona Stanley Hospital, Perth	Princess Marina Hospital, Gaborone	Haaglanden Medical Centre Westeinde, The Hague
**EHI_A thresholds**	-1.6; -1.3; 2.4	-2.3; -1.5; -1.4	N/A	N/A	N/A

The current study did not identify a threshold temperature associated with particularly large increases in ED demand and this means that most of the temperature-ED relationships shown in Fig 2 are shown as a straight line.

## Discussion

Consistent with prior studies [12], the current study shows an association between ambient temperature and morbidity. In each of the regions, increases in temperature that do not coincide with time to acclimatise result in increases in the number of ED attendances. In our study, this increase was strongest for the Aga Khan University Hospital in Karachi. This may be due to various factors, including the hospital’s large catchment area and good reputation in the region. Problems with ED demand during extreme heat are not unknown to this area. For example, a severe heatwave in Karachi in July 2015 resulted in more than 1,200 deaths in 10 days [18]. It should be noted that, despite its enormous impact and high temperatures (up to 44.8 °C in Karachi), this heatwave did not result in any day with EHIaccl above 7. Although it was characterised by multiple extremely hot days in a row, the maximum EHIaccl in these days was only 4.9.

The impact of this heatwave in Pakistan was likely exacerbated due to low wind, high humidity, frequent power outages which crippled the city’s water supply system, the Islamic holy month of Ramadan (during which the majority of Muslims observe fasting of ~15 hours), and a poor response by the local authorities [18]. Unfortunately, such effects were not considered in the current study due to data limitations. Another limitation of the data was the lack of information on air pollution. Air pollution has not been controlled for in the current study, even though it affects cardiovascular and respiratory morbidities [12, 15]. The same is true for droughts. While droughts are known to increase the risk of mortality [19], their effects (either independently or during heatwaves) were not considered in this study.

The association between heat and ED demand differed between regions. Hot days were associated with a higher proportion of ED visits by young children in Sacramento and the Netherlands, possibly related to school holidays. Another explanation may be the higher risk of dehydration and electrolyte imbalance in children [15], and the fact that exertional heat stroke primarily affects younger active populations and can develop within hours [20]. Our study might have missed the effect of classic heat strokes since these usually develop gradually over several days with minimally raised core temperature, which is more common in elderly patients. Delayed effects were not accounted for in the current study. As opposed to the Western regions, the hospital in Karachi did have a higher proportion of elderly coming in to ED on hot days. The elderly are known to be more prone to heat-related illness.

In contrast to earlier findings, we did not show more visits/admissions/deaths for categories of symptoms which have often been associated with heat. The only patients who were more likely to die during hot days (EHIaccl ≥ 4) were patients with psychiatric symptoms in The Hague, possibly due to self-inflicted harm. Prior research has shown an association between increasing mean apparent temperature with mental health outcomes and intentional injuries [21]. Unfortunately, diagnostic data was not available for the current study. While we did have data about presenting complaints, we do not know how many or which patients were diagnosed with heat-related illnesses.

The current study shows that in all regions, the EHI_A indicator has an average that is close to zero and closely coincides with a normal distribution. The EHI_A performs in a similar manner across different climates. We did not identify a threshold temperature associated with particularly large increases in ED demand. More advanced threshold models or panel data techniques might provide more insight into the types of climate conditions that would warrant governmental or healthcare provider interventions. Such models will also benefit from taking into account lagged health effects, e.g. effects that are delayed by up to several days [9, 12]. Not considering these effects in our model may have resulted in omitted variable bias, the direction of which is unknown.

Heat is a problem in each of the included regions. In Australia, heatwaves have been recognised as the nation’s most deadly natural hazard, causing 55% of all natural disaster-related deaths and burdening the Australian workforce by approximately $6.2 billion every year [22]. In the Netherlands, the health effect of high temperatures under a changing climate has been identified by the Health Council of The Netherlands and the Dutch Court of Audits as an important issue demanding further research and policy action, however the Dutch mortality and morbidity impacts during heat events have rarely been studied before [23]. In the USA, in addition to resulting in mortality and morbidity, heatwaves contributed over five billion dollars of health costs during 2000–2009. A recent national survey found that response plans for extreme hot temperatures were far from adequate in the USA [24]. Based on climate projections for different cities in California, the number of heatwave days (defined as 3 or more consecutive days with a temperature above 32°C) in Sacramento will increase from 58 (1961–1990) to 109–138 (2070–2099), dependent on the climate model and emissions scenario [25]. In Botswana, heat-related deaths in the elderly (65+ years) are projected to increase from approximately 3 to 136 per 100,000 by 2080 [26].

While many global cities expect climate change, including extreme temperatures, to seriously compromise public health infrastructures, spending for climate change adaptation remains minimal [27]. Given the association between heat and ED use, hospitals and governmental authorities should evaluate the demands that heat can place on their local health care systems. These demands may differ substantially between regions. Associations between heat and ED use suggest that the demand for emergency services in Karachi may be most heavily affected by sudden, high temperatures.

## Supporting information

S1 Table Hospital admissions1 and patient/visit characteristics on hot days versus other days. (DOCX)

S2 Table Mortality and patient/visit characteristics on hot days versus other days. (DOCX)

S3 Table Threshold regression estimates—EHI_A. (DOCX)

S1 Fig Histograms of the acclimatisation excess heat index (EHI_A) by location, for five hospitals in different regions. (DOCX)

S2 Fig Mean number of ED attendances dependent on different exposure measures. (DOCX)
