# Supplementary material for: The use of an ‘acclimatisation’ heatwave measure to compare temperature-related demand for emergency services in Australia, Botswana, Netherlands, Pakistan, and USA
Source: PLoS One. 2019 Mar 28;14(3):e0214242. doi: 10.1371/journal.pone.0214242 (PMC6438466; doi:10.1371/journal.pone.0214242)
Supplement: S1 Table — (DOCX) [file pone.0214242.s001.docx]

**S1 Table. Hospital admissions^1^ and patient/visit characteristics on hot days versus other days.**

|  | **Hot days, EHIaccl ≥ 4** | **Other days, EHIaccl < 4** | **Sig^2^** | **Hot days, EHIaccl ≥ 7** | **Other days, EHIaccl < 7** | **Sig^1^** |
| --- | --- | --- | --- | --- | --- | --- |
| **Netherlands, The Hague (3 locations)** | | | | | | |
| N | 191 | 2,690 |  | 22 | 2,859 |  |
| Age, mean % (sd) |  |  |  |  |  |  |
| <5 | 6.8 (13.3) | 9.6 (18.7) | 0.047* | 5.7 (3.8) | 9.5 (18.4) | 0.338 |
| 5-11 | 3.8 (10.9) | 3.3 (9.8) | 0.566 | 1.4 (1.6) | 3.4 (9.9) | 0.345 |
| 12-17 | 1.9 (3.4) | 2.4 (7.2) | 0.378 | 1.5 (1.9) | 2.4 (7.0) | 0.567 |
| 18-34 | 9.9 (6.4) | 9.7 (7.7) | 0.718 | 8.9 (4.4) | 9.7 (7.7) | 0.613 |
| 35-64 | 33.3 (11.1) | 32.4 (13.6) | 0.355 | 32.7 (6.9) | 32.4 (13.5) | 0.923 |
| 65-74 | 16.9 (9.3) | 15.8 (8.8) | 0.076 | 18.5 (5.6) | 15.8 (8.8) | 0.162 |
| 75-84 | 16.9 (7.5) | 16.3 (8.8) | 0.354 | 20.3 (5.9) | 16.3 (8.7) | 0.035* |
| 85+ | 10.3 (5.8) | 10.5 (7.0) | 0.768 | 11.0 (6.3) | 10.4 (6.9) | 0.691 |
| Proportion of males, mean (sd) | 50.2 (11.6) | 51.5 (14.5) | 0.258 | 50.9 (9.6) | 51.4 (14.3) | 0.878 |
| Acuity, mean % (sd) |  |  |  |  |  |  |
| Non-urgent (blue, green) | 11.5 (9.1) | 11.4 (12.1) | 0.919 | 8.9 (4.4) | 11.5 (11.9) | 0.307 |
| Urgent (yellow, orange, red) | 76.3 (16.4) | 76.0 (16.9) | 0.847 | 78.0 (17.4) | 76.0 (16.8) | 0.580 |
| Not triaged | 12.2 (15.0) | 12.5 (14.0) | 0.752 | 13.1 (15.7) | 12.5 (14.1) | 0.840 |
| Presenting complaints |  |  |  |  |  |  |
| Cardiac | 7.4 (6.3) | 9.2 (6.3) | 0.002* | 9.5 (5.6) | 9.1 (6.3) | 0.795 |
| Diabetes Mellitus | 0.5 (0.9) | 0.5 (1.1) | 0.580 | 0.7 (0.9) | 0.5 (1.0) | 0.520 |
| Malaise | 7.7 (7.3) | 9.0 (6.7) | 0.037* | 10.1 (7.2) | 8.9 (6.8) | 0.484 |
| Psychiatric | 1.9 (2.5) | 2.2 (2.4) | 0.219 | 1.3 (1.5) | 2.2 (2.4) | 0.139 |
| Renal/urinary | 0.9 (1.5) | 1.1 (1.6) | 0.086 | 0.7 (1.1) | 1.1 (1.6) | 0.245 |
| Respiratory | 7.9 (6.9) | 9.5 (6.8) | 0.015* | 11.6 (6.4) | 9.3 (6.8) | 0.184 |
| **Pakistan** | | | | | | |
| N | 53 | 1766 |  | 1 | 1,818 |  |
| Age, mean % (sd) |  |  |  |  |  |  |
| <5 | 17.5 (7.6) | 18.6 (6.9) | 0.249 | 9.8 (-) | 18.6 (6.9) | 0.206 |
| 5-11 | 6.4 (4.0) | 6.8 (4.2) | 0.521 | 5.9 (-) | 6.8 (4.2) | 0.826 |
| 12-17 | 3.6 (2.8) | 4.4 (3.5) | 0.108 | 7.8 (-) | 4.3 (3.5) | 0.320 |
| 18-34 | 15.2 (5.9) | 15.8 (6.2) | 0.526 | 19.6 (-) | 15.8 (6.2) | 0.532 |
| 35-64 | 33.3 (8.2) | 32.5 (8.8) | 0.546 | 33.3 (-) | 32.6 (8.8) | 0.929 |
| 65-74 | 14.0 (6.6) | 12.4 (5.6) | 0.038* | 13.7 (-) | 12.5 (5.6) | 0.823 |
| 75-84 | 8.2 (4.5) | 7.5 (4.4) | 0.289 | 7.8 (-) | 7.6 (4.4) | 0.950 |
| 85+ | 1.8 (2.0) | 2.0 (3.3) | 0.664 | 2.0 (-) | 2.0 (3.2) | 0.999 |
| Proportion of males, mean (sd) | 55.7 (10.1) | 55.9 (8.7) | 0.825 | 2.0 (-) | 55.9 (8.8) | 0.574 |
| Acuity, mean % (sd) |  |  |  |  |  |  |
| Non-urgent (blue, green) | 4.0 (5.1) | 4.0 (5.8) | 0.982 | 2.0 (-) | 4.0 (5.8) | 0.723 |
| Urgent (yellow, orange, red) | 96.0 (5.1) | 96.0 (5.8) | 0.982 | 98.0 (-) | 96.0 (5.8) | 0.723 |
| Not triaged | 0.0 (0.0) | 0.0 (0.0) | - | 0.0 (0.0) | 0.0 (0.0) | - |
| **USA** | | | | | | |
| N | 176 | 2,432 |  | 15 | 2,593 |  |
| Age, mean % (sd) |  |  |  |  |  |  |
| <5 | 6.3 (12.6) | 7.2 (17.8) | 0.533 | 6.1 (12.0) | 7.2 (17.6) | 0.815 |
| 5-11 | 5.5 (16.3) | 3.3 (10.9) | 0.013* | 1.7 (6.5) | 3.5 (11.4) | 0.545 |
| 12-17 | 3.3 (10.7) | 3.6 (12.1) | 0.742 | 2.2 (8.6) | 3.6 (12.0) | 0.667 |
| 18-34 | 15.2 (20.9) | 14.9 (22.0) | 0.853 | 17.4 (14.9) | 14.9 (22.0) | 0.662 |
| 35-64 | 45.6 (30.0) | 43.0 (31.4) | 0.286 | 58.5 (27.3) | 43.1 (31.3) | 0.056 |
| 65-74 | 11.1 (18.6) | 12.1 (20.7) | 0.538 | 6.8 (13.2) | 12.1 (20.6) | 0.318 |
| 75-84 | 7.1 (14.3) | 9.8 (19.1) | 0.062 | 1.9 (4.1) | 9.7 (18.8) | 0.112 |
| 85+ | 5.8 (13.3) | 6.1 (15.4) | 0.839 | 5.3 (9.9) | 6.1 (15.3) | 0.857 |
| Proportion of males, mean (sd) | 55.3 (27.2) | 53.3 (31.5) | 0.428 | 42.7 (28.4) | 53.5 (31.2) | 0.179 |
| Acuity, mean % (sd) |  |  |  |  |  |  |
| Non-urgent (blue, green) | 2.2 (8.7) | 1.4 (6.9) | 0.137 | 1.0 (3.7) | 1.5 (7.1) | 0.780 |
| Urgent (yellow, orange, red) | 97.1 (9.6) | 97.3 (10.1) | 0.790 | 99.0 (3.7) | 97.3 (10.0) | 0.496 |
| Not triaged | 0.7 (3.7) | 1.3 (7.3) | 0.271 | 0.0 (0.0) | 1.3 (7.2) | 0.497 |
| Presenting complaints |  |  |  |  |  |  |
| Cardiac | 10.7 (20.7) | 9.1 (17.6) | 0.239 | 11.0 (25.9) | 9.2 (17.8) | 0.688 |
| Dehydration | 0.0 (0.0) | 0.1 (1.7) | 0.304 | 0.0 (0.0) | 0.1 (1.7) | 0.771 |
| Diabetes Mellitus | 0.3 (2.3) | 0.6 (3.5) | 0.302 | 0.0 (0.0) | 0.5 (3.4) | 0.537 |
| Heat exhaustion | 0.0 (0.0) | 0.0 (0.4) | 0.788 | 0.0 (0.0) | 0.0 (0.4) | 0.939 |
| Malaise | 0.0 (0.0) | 0.0 (0.4) | 0.706 | 0.0 (0.0) | 0.0 (0.4) | 0.915 |
| Psychiatric | 4.3 (10.2) | 5.2 (13.3) | 0.399 | 2.4 (6.5) | 5.1 (13.1) | 0.433 |
| Renal/urinary | 0.8 (5.7) | 0.3 (3.0) | 0.020* | 0.5 (2.1) | 0.3 (3.2) | 0.770 |
| Respiratory | 2.2 (6.7) | 3.8 (12.5) | 0.086 | 1.2 (3.8) | 3.7 (12.2) | 0.429 |
| Stroke | 1.2 (4.8) | 1.7 (7.8) | 0.428 | 0.3 (1.0) | 1.6 (7.6) | 0.486 |

^1^ These only include hospital admissions that were made through the emergency department.

^2^ 2-tailed, equal variances assumed.

^3^ This data was only available for part of the period (from 01/01/2014 onwards). The number of hot days in this period was 10 (EHIaccl ≥ 7) or 66 (EHIaccl ≥ 4).
