# Supplementary material for: The use of an ‘acclimatisation’ heatwave measure to compare temperature-related demand for emergency services in Australia, Botswana, Netherlands, Pakistan, and USA
Source: PLoS One. 2019 Mar 28;14(3):e0214242. doi: 10.1371/journal.pone.0214242 (PMC6438466; doi:10.1371/journal.pone.0214242)
Supplement: S2 Table — (DOCX) [file pone.0214242.s002.docx]

**S2 Table. Mortality^1^ and patient/visit characteristics on hot days versus other days.**

|  | **Hot days, EHIaccl ≥ 4** | **Other days, EHIaccl < 4** | **Sig^2^** | **Hot days, EHIaccl ≥ 7** | **Other days, EHIaccl < 7** | **Sig^1^** |
| --- | --- | --- | --- | --- | --- | --- |
| **Netherlands, The Hague (3 locations)** | | | | | | |
| N | 34 | 476 |  | 2 | 508 |  |
| Age, mean % (sd) |  |  |  |  |  |  |
| <5 | 0.0 (0.0) | 0.2 (4.6) | 0.790 | 0.0 (0.0) | 0.2 (4.4) | 0.950 |
| 5-11 | 0.0 (0.0) | 0.0 (0.0) | - | 0.0 (0.0) | 0.0 (0.0) | - |
| 12-17 | 0.0 (0.0) | 0.8 (9.1) | 0.592 | 0.0 (0.0) | 0.8 (8.8) | 0.900 |
| 18-34 | 5.9 (19.2) | 4.4 (19.5) | 0.671 | 0.0 (0.0) | 4.5 (19.5) | 0.743 |
| 35-64 | 35.3 (48.5) | 30.5 (44.6) | 0.544 | 0.0 (0.0) | 30.9 (44.9) | 0.331 |
| 65-74 | 20.6 (41.0) | 22.3 (40.3) | 0.814 | 0.0 (0.0) | 22.2 (40.3) | 0.436 |
| 75-84 | 20.6 (41.0) | 24.1 (40.9) | 0.633 | 100 (0.0) | 23.5 (40.7) | 0.008* |
| 85+ | 17.6 (36.0) | 17.8 (35.5) | 0.987 | 0.0 (0.0) | 17.8 (35.6) | 0.479 |
| Proportion of males, mean (sd) | 64.7 (46.4) | 59.9 (47.3) | 0.565 | 0.0 (0.0) | 60.4 (47.1) | 0.071 |
| Acuity, mean % (sd) |  |  |  |  |  |  |
| Non-urgent (blue, green) | 2.9 (17.1) | 1.3 (10.2) | 0.380 | 0.0 (0.0) | 1.4 (10.8) | 0.857 |
| Urgent (yellow, orange, red) | 91.2 (28.8) | 87.8 (29.8) | 0.524 | 100 (0.0) | 88.0 (29.7) | 0.569 |
| Not triaged | 5.9 (23.9) | 10.9 (28.5) | 0.314 | 0.0 (0.0) | 10.6 (28.2) | 0.595 |
| Presenting complaints |  |  |  |  |  |  |
| Cardiac | 5.3 (22.9) | 11.1 (30.5) | 0.414 | 50.0 (70.7) | 10.4 (29.6) | 0.063 |
| Diabetes Mellitus | 0.0 (0.0) | 0.0 (0.0) | - | 0.0 (0.0) | 0.0 (0.0) | - |
| Malaise | 5.3 (22.9) | 9.3 (26.2) | 0.520 | 0.0 (0.0) | 9.0 (26.1) | 0.625 |
| Psychiatric | 5.3 (15.8) | 0.4 (4.5) | 0.001* | 0.0 (0.0) | 0.8 (6.2) | 0.860 |
| Renal/urinary | 0.0 (0.0) | 0.0 (0.0) | - | 0.0 (0.0) | 0.0 (0.0) | - |
| Respiratory | 26.3 (45.2) | 11.5 (31.3) | 0.057 | 50.0 (70.7) | 12.3 (32.3) | 0.104 |
| **Pakistan** | | | | | | |
| N | 39 | 1,382 |  | 1 | 1,420 |  |
| Age, mean % (sd) |  |  |  |  |  |  |
| <5 | 8.2 (20.6) | 7.2 (20.4) | 0.769 | 0.0 (-) | 59.7 (37.9) | 0.722 |
| 5-11 | 3.8 (17.7) | 1.4 (8.8) | 0.103 | 0.0 (-) | 7.3 (20.4) | 0.869 |
| 12-17 | 1.3 (8.0) | 1.7 (10.3) | 0.781 | 0.0 (-) | 1.5 (9.1) | 0.866 |
| 18-34 | 9.6 (19.3) | 10.8 (23.6) | 0.739 | 0.0 (-) | 1.7 (10.2) | 0.646 |
| 35-64 | 37.8 (35.6) | 39.3 (38.0) | 0.806 | 0.0 (-) | 10.8 (23.5) | 0.301 |
| 65-74 | 11.2 (22.1) | 19.7 (30.8) | 0.085 | 0.0 (-) | 39.3 (38.0) | 0.524 |
| 75-84 | 20.9 (32.5) | 15.0 (27.4) | 0.188 | 50.0 (-) | 19.4 (30.6) | 0.206 |
| 85+ | 7.2 (19.3) | 4.7 (15.9) | 0.331 | 50.0 (-) | 15.1 (27.5) | 0.005* |
| Proportion of males, mean (sd) | 65.2 (38.2) | 59.5 (37.8) | 0.356 | 50.0 (-) | 4.7 (16.0) | 0.798 |
| Acuity, mean % (sd) |  |  |  |  |  |  |
| Non-urgent (blue, green) | 5.2 (18.5) | 4.3 (16.6) | 0.738 | 0.0 (-) | 4.3 (16.6) | 0.794 |
| Urgent (yellow, orange, red) | 94.8 (18.5) | 95.7 (16.6) | 0.738 | 100.0 (-) | 95.7 (16.6) | 0.794 |
| Not triaged | 0.0 (0.0) | 0.0 (0.0) | - | 0.0 (-) | 0.0 (-) | - |
| **USA** | | | | | | |
| N | 59 | 923 |  | 8 | 974 |  |
| Age, mean % (sd) |  |  |  |  |  |  |
| <5 | 3.4 (18.3) | 7.5 (25.5) | 0.219 | 12.5 (35.4) | 7.2 (25.1) | 0.556 |
| 5-11 | 1.7 (13.0) | 1.2 (10.1) | 0.736 | 0.0 (0.0) | 1.3 (10.4) | 0.730 |
| 12-17 | 0.8 (6.5) | 1.1 (9.6) | 0.830 | 0.0 (0.0) | 1.1 (9.5) | 0.740 |
| 18-34 | 16.1 (35.3) | 11.3 (29.5) | 0.233 | 25.0 (46.3) | 11.5 (29.7) | 0.203 |
| 35-64 | 39.8 (46.2) | 37.6 (45.8) | 0.721 | 37.5 (51.8) | 37.8 (45.8) | 0.987 |
| 65-74 | 14.4 (34.8) | 13.3 (31.8) | 0.788 | 12.5 (35.4) | 13.3 (32.0) | 0.942 |
| 75-84 | 15.3 (32.5) | 13.7 (32.6) | 0.718 | 0.0 (0.0) | 13.9 (32.7) | 0.231 |
| 85+ | 8.5 (28.1) | 14.2 (33.6) | 0.197 | 12.5 (35.4) | 13.9 (33.3) | 0.906 |
| Proportion of males, mean (sd) | 54.2 (49.4) | 63.3 (45.5) | 0.139 | 37.5 (51.8) | 63.0 (45.7) | 0.116 |
| Acuity, mean % (sd) |  |  |  |  |  |  |
| Non-urgent (blue, green) | 0.0 (0.0) | 0.0 (0.0) | - | 0.0 (0.0) | 0.0 (0.0) | - |
| Urgent (yellow, orange, red) | 91.5 (28.1) | 92.9 (24.6) | 0.688 | 75.0 (46.3) | 92.9 (24.5) | 0.041* |
| Not triaged | 8.5 (28.0 | 7.1 (24.6) | 0.688 | 25.0 (46.3) | 7.1 (24.5) | 0.041* |
| Presenting complaints |  |  |  |  |  |  |
| Cardiac | 3.4 (18.3) | 1.9 (12.9) | 0.409 | 12.5 (35.4) | 1.9 (13.0) | 0.025* |
| Dehydration | 0.0 (0.0) | 0.0 (0.0) | - | 0.0 (0.0) | 0.0 (0.0) | - |
| Diabetes Mellitus | 0.0 (0.0) | 0.1 (1.6) | 0.801 | 0.0 (0.0) | 0.1 (1.6) | 0.928 |
| Heat exhaustion | 0.0 (0.0) | 0.0 (0.0) | - | 0.0 (0.0) | 0.0 (0.0) | - |
| Malaise | 0.0 (0.0) | 0.0 (0.0) | - | 0.0 (0.0) | 0.0 (0.0) | - |
| Psychiatric | 5.9 (20.9) | 7.6 (25.5) | 0.626 | 12.5 (35.4) | 7.4 (25.2) | 0.573 |
| Renal/urinary | 0.0 (0.0) | 0.0 (0.0) | - | 0.0 (0.0) | 0.0 (0.0) | - |
| Respiratory | 0.0 (0.0) | 0.9 (8.8) | 0.415 | 0.0 (0.0) | 0.9 (8.6) | 0.770 |
| Stroke | 0.0 (0.0) | 0.6 (7.5) | 0.543 | 0.0 (0.0) | 0.6 (7.3) | 0.827 |

^1^ This only includes mortality of patients who arrived through the emergency department.

^2^ 2-tailed, equal variances assumed.

^3^ This data was only available for part of the period (from 01/01/2014 onwards). The number of hot days in this period was 10 (EHIaccl ≥ 7) or 66 (EHIaccl ≥ 4).
