# Supplementary material for: The use of an ‘acclimatisation’ heatwave measure to compare temperature-related demand for emergency services in Australia, Botswana, Netherlands, Pakistan, and USA
Source: PLoS One. 2019 Mar 28;14(3):e0214242. doi: 10.1371/journal.pone.0214242 (PMC6438466; doi:10.1371/journal.pone.0214242)
Supplement: S3 Table — (DOCX) [file pone.0214242.s003.docx]

**S3 Table. Threshold regression estimates – EHI_A.**

|  | **Aga Khan** | **Davis** | **Fiona Stanley** | **Princess Marina** | **Haaglanden-**  **Westeinde** |
| --- | --- | --- | --- | --- | --- |
| **Mon** | 11.14*** | 14.89*** | 23.37*** | 1.099 | 12.66*** |
|  | (2.327) | (1.142) | (2.822) | (1.726) | (0.905) |
| **Tue** | -0.639 | 3.099*** | 0.914 | 0.528 | 2.861*** |
|  | (2.326) | (1.142) | (2.813) | (1.732) | (0.905) |
| **Thu** | -1.340 | -3.074*** | -3.293 | -0.798 | 1.137 |
|  | (2.325) | (1.142) | (2.812) | (1.732) | (0.905) |
| **Fri** | -0.870 | -0.248 | 0.603 | 2.235 | 10.33*** |
|  | (2.325) | (1.142) | (2.814) | (1.726) | (0.905) |
| **Sat** | 8.292*** | -11.09*** | 13.74*** | -4.734*** | 8.753*** |
|  | (2.325) | (1.142) | (2.816) | (1.726) | (0.904) |
| **Sun** | 32.90*** | -14.89*** | 32.81*** | -4.886*** | 8.456*** |
|  | (2.327) | (1.142) | (2.827) | (1.727) | (0.905) |
| **Jan** | -19.07*** | -3.625** | -3.008 | -4.245* | -4.417*** |
|  | (3.108) | (1.535) | (4.565) | (2.532) | (1.231) |
| **Feb** | -17.36*** | 0.369 | -2.648 | 1.807 | -6.598*** |
|  | (3.046) | (1.512) | (3.731) | (2.664) | (1.199) |
| **Mar** | -18.40*** | -1.600 | 6.612* | 0.665 | -4.055*** |
|  | (3.153) | (1.475) | (3.593) | (2.576) | (1.168) |
| **Apr** | -10.03*** | -2.495* | -9.549*** | 4.169 | 0.805 |
|  | (2.999) | (1.486) | (3.603) | (2.569) | (1.178) |
| **Jun** | 2.535 | -5.884*** | 2.808 | -4.956* | 2.772** |
|  | (2.987) | (1.490) | (3.646) | (2.559) | (1.177) |
| **Jul** | 16.29*** | -6.663*** | -10.83*** | -5.903** | -6.354*** |
|  | (3.076) | (1.479) | (3.616) | (2.552) | (1.168) |
| **Aug** | 18.13*** | -5.685*** | 5.669 | -2.435 | -5.864*** |
|  | (3.030) | (1.496) | (3.679) | (2.745) | (1.175) |
| **Sep** | 12.72*** | -3.028** | 2.058 | -0.898 | 2.659** |
|  | (3.031) | (1.516) | (3.707) | (2.718) | (1.203) |
| **Oct** | -4.192 | -9.224*** | -4.624 | 3.116 | -2.077* |
|  | (3.079) | (1.561) | (3.787) | (2.459) | (1.211) |
| **Nov** | 1.817 | -9.906*** | 2.005 | 3.108 | -3.668*** |
|  | (3.468) | (1.627) | (3.889) | (2.320) | (1.221) |
| **Dec** | -9.623*** | -12.03*** | -0.300 | -2.377 | -4.986*** |
|  | (3.685) | (1.522) | (3.708) | (2.414) | (1.188) |
| **Year_2** | 33.11*** | -10.73*** |  |  | 0.195 |
|  | (2.494) | (1.245) |  |  | (0.987) |
| **Year_3** | 32.05*** | 12.95*** |  |  | 6.059*** |
|  | (2.488) | (1.246) |  |  | (0.987) |
| **Year_4** | 70.85*** | 22.61*** |  |  | 4.339*** |
|  | (2.490) | (1.244) |  |  | (0.986) |
| **Year_5** | 86.55*** | 20.44*** |  |  | 0.124 |
|  | (2.494) | (1.246) |  |  | (0.987) |
| **Year_6** | 102.6*** | 38.78*** |  |  | 2.252** |
|  | (2.559) | (1.246) |  |  | (0.987) |
| **Year_7** | 88.95*** | 49.33*** | -3.011* | 0.406 | 3.626*** |
|  | (2.502) | (1.245) | (1.540) | (1.392) | (0.987) |
| **Year_8** | 137.2*** | 52.11*** |  |  | 6.542*** |
|  | (2.670) | (1.245) |  |  | (0.986) |
| **EHI_A (Region 1)** | 0.561 | 1.318*** | 1.214*** | 1.240*** | 1.307*** |
|  | (0.777) | (0.215) | (0.360) | (0.220) | (0.101) |
| **EHI_A (Region 2)** | -9.565*** | 3.234*** |  |  |  |
|  | (2.515) | (0.608) |  |  |  |
| **EHI_A (Region 3)** | -0.669 | -4.273** |  |  |  |
|  | (0.915) | (2.024) |  |  |  |
| **EHI_A (Region 4)** | 1.544** | 0.861*** |  |  |  |
|  | (0.693) | (0.206) |  |  |  |
| **Constant** | 84.72*** | 156.2*** | 247.3*** | 47.74*** | 119.4*** |
|  | (3.125) | (2.645) | (6.789) | (3.735) | (1.794) |
| **Observations** | 2,782 | 2,922 | 697 | 489 | 2,922 |

Note: Statistical significance is indicated as *** p<0.01, ** p<0.05, * p<0.1. When variables are non-significant, the impact of these variables on the number of ED attendances is not significantly different from the reference category.

Abbreviations: EHI_A, acclimatisation excess heat index.

Wednesday, May and Year_1 are the reference categories.
