# Supplementary material for: The use of an ‘acclimatisation’ heatwave measure to compare temperature-related demand for emergency services in Australia, Botswana, Netherlands, Pakistan, and USA
Source: PLoS One. 2019 Mar 28;14(3):e0214242. doi: 10.1371/journal.pone.0214242 (PMC6438466; doi:10.1371/journal.pone.0214242)
Supplement: S1 Fig — (DOCX) [file pone.0214242.s004.docx]

**S1 Fig. Histograms of the acclimatisation Excess Heat Index (EHI_A) by location, for five hospitals in different regions.**

Aga Khan: UC Davis:

Fiona Stanley: Princess Marina:

HMC Westeinde:
