# Supplementary material for: The use of an ‘acclimatisation’ heatwave measure to compare temperature-related demand for emergency services in Australia, Botswana, Netherlands, Pakistan, and USA
Source: PLoS One. 2019 Mar 28;14(3):e0214242. doi: 10.1371/journal.pone.0214242 (PMC6438466; doi:10.1371/journal.pone.0214242)
Supplement: S2 Fig — (DOCX) [file pone.0214242.s005.docx]

**S2 Fig. Mean number of ED attendances dependent on different exposure measures.**

|  | **Mean number of ED attendances dependent on the maximum temperature in °C on that day** | **Mean number of ED attendances dependent on the three daily maximum temperature** | **Mean number of ED attendances dependent on the three daily average temperature** |
| --- | --- | --- | --- |
| **Australia** | 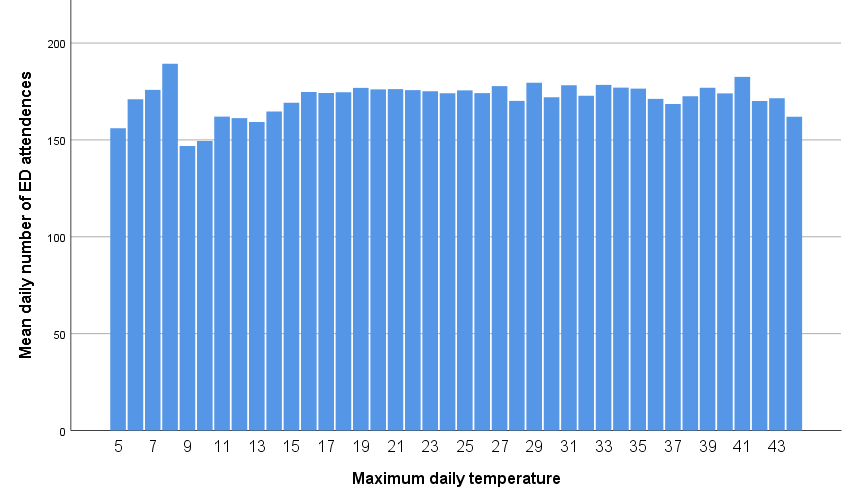 | 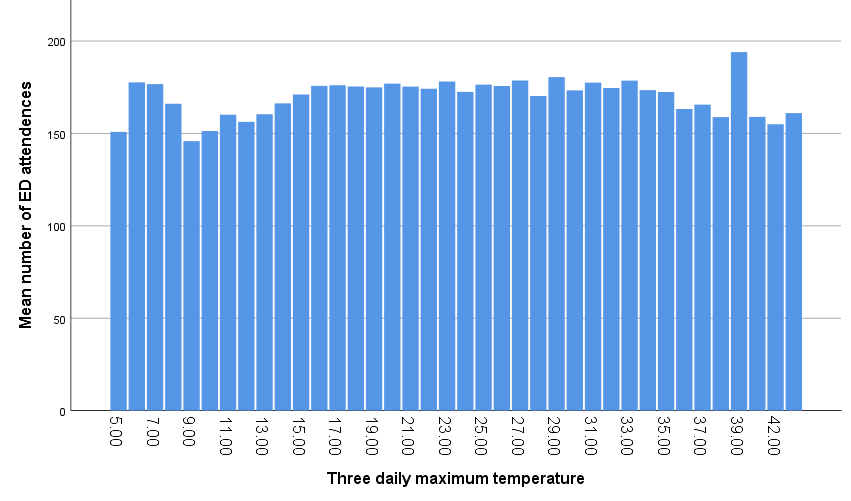 | 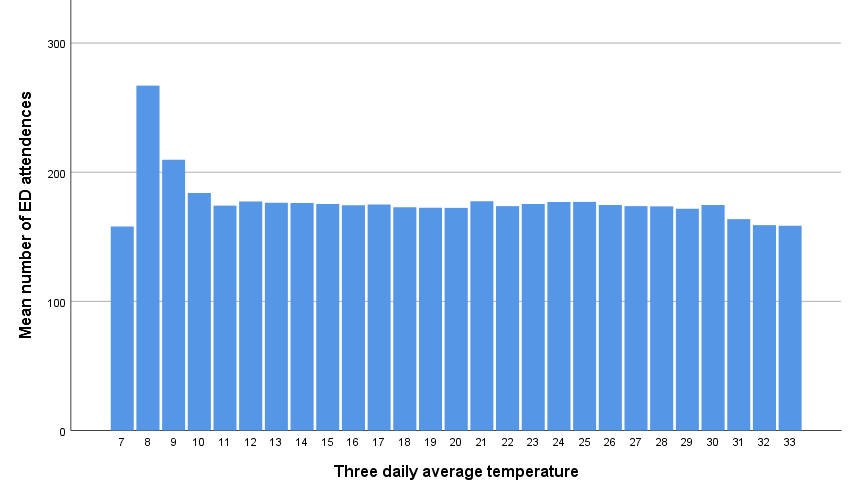 |
| **Botswana** | 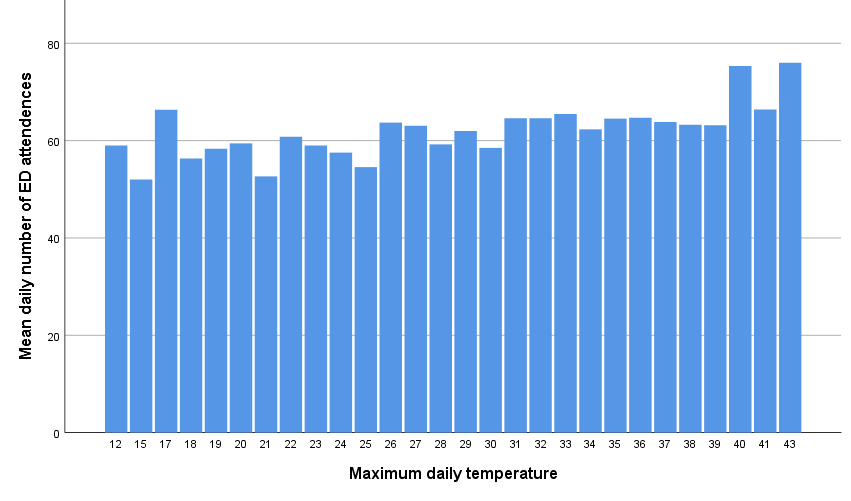 | 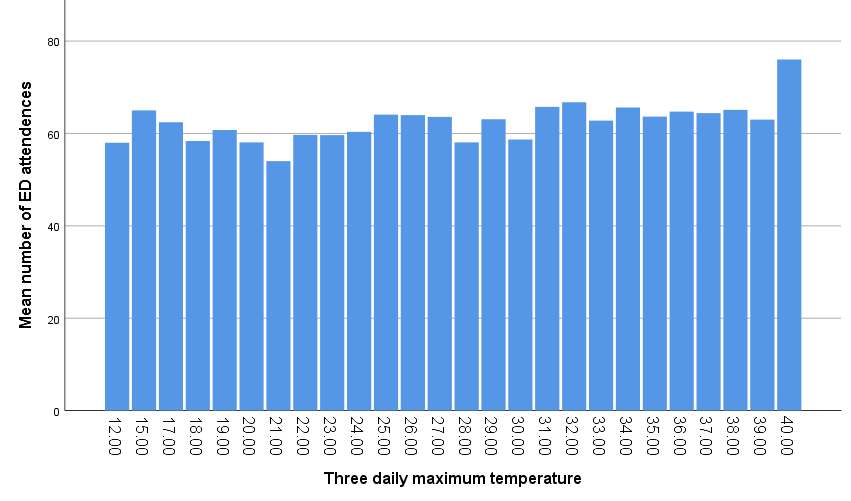 | 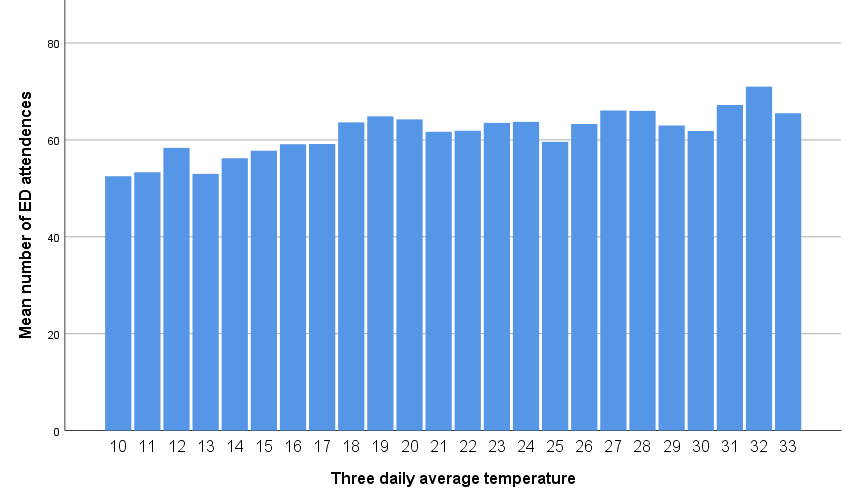 |
| **Netherlands** | 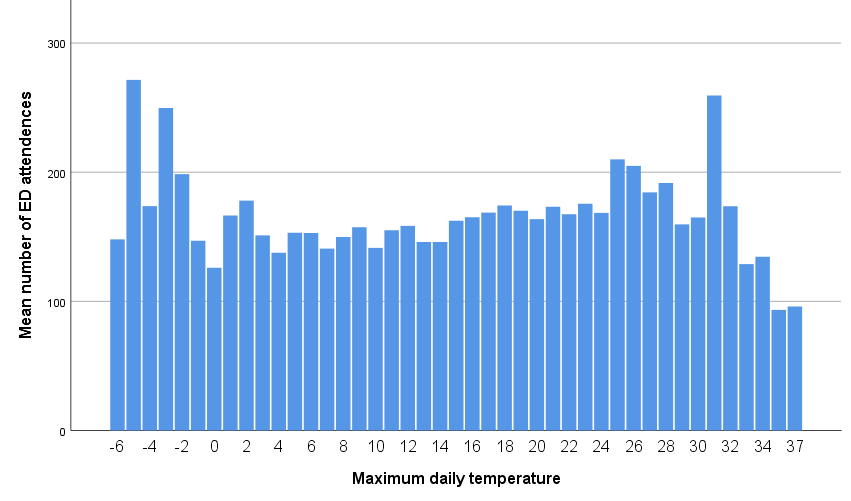 | 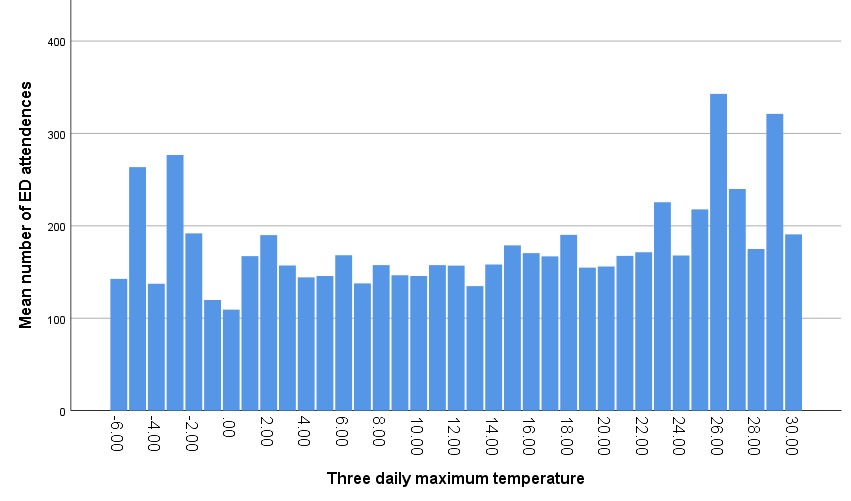 | 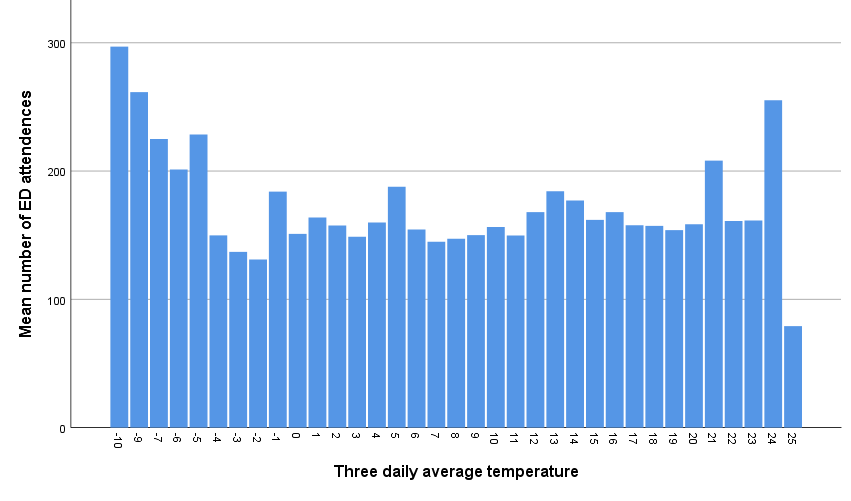 |
| **Pakistan** | 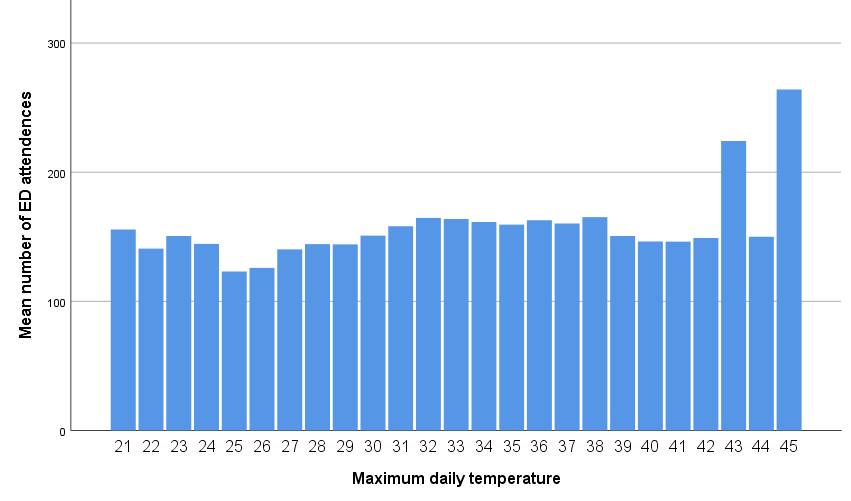 | 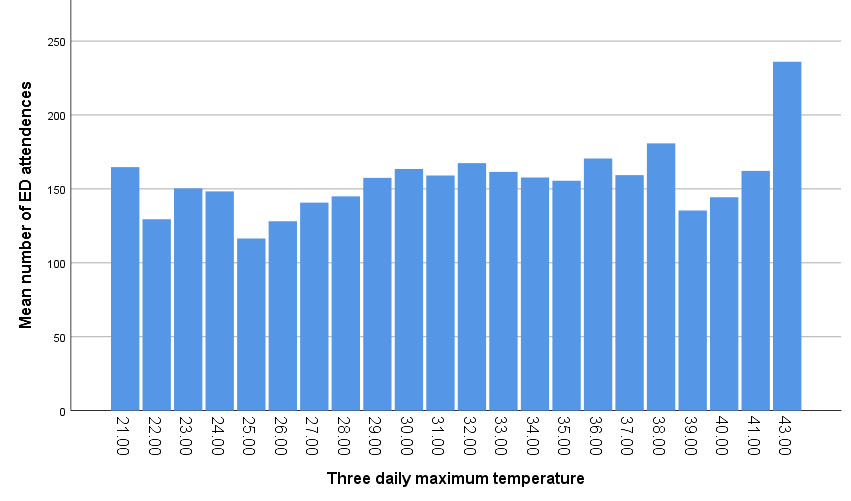 | 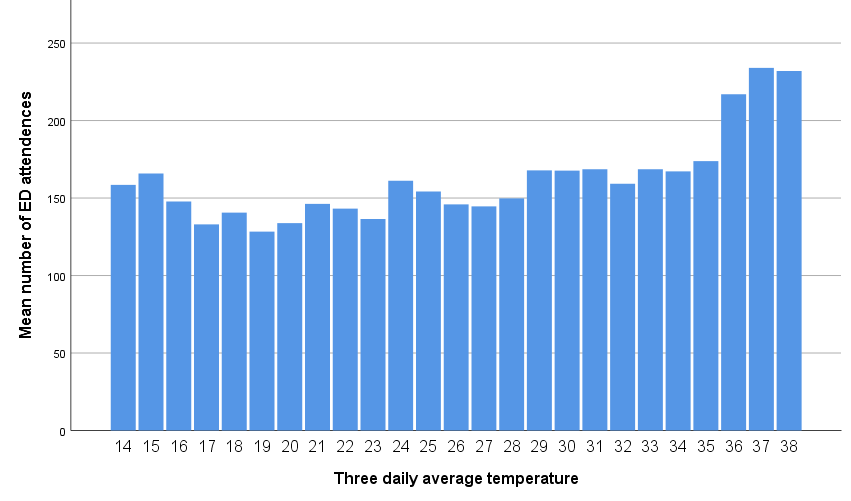 |
| **USA** | 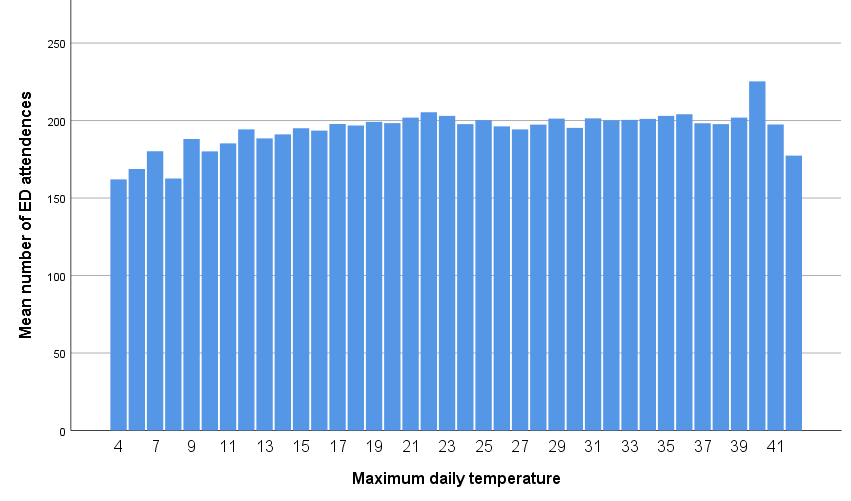 | 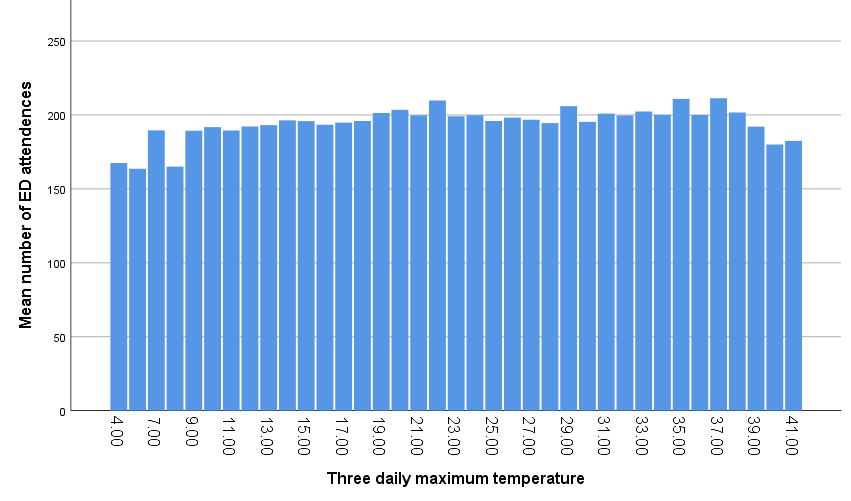 | 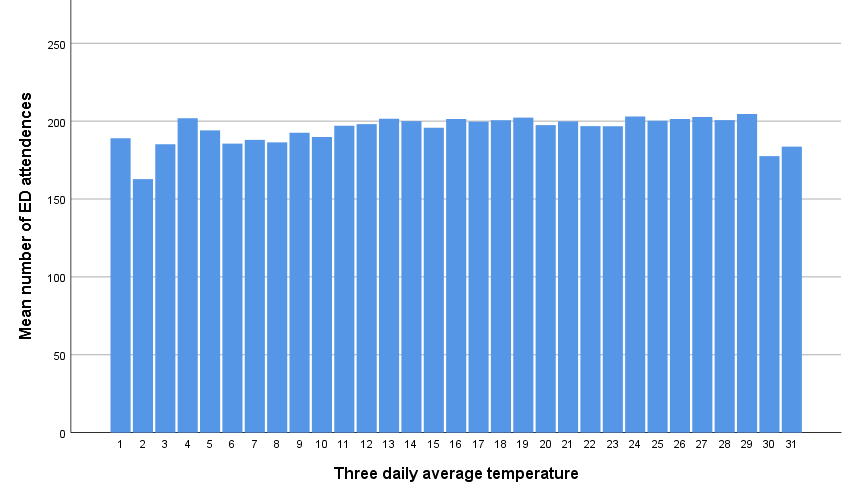 |

|  | **Mean number of ED attendances dependent on the significant excess heat index** | **Mean number of ED attendances dependent on the acclimatisation Excess Heat Index** | **Mean number of ED attendances dependent on the excess heat factor** |
| --- | --- | --- | --- |
| **Australia** | 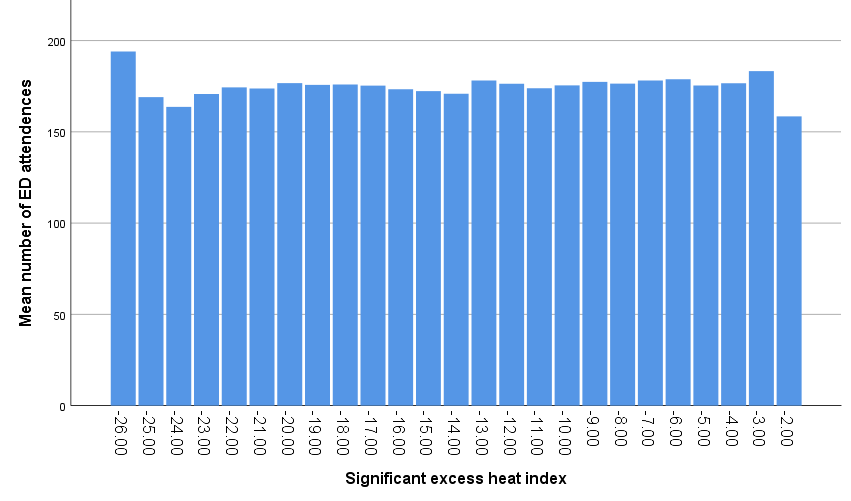 | 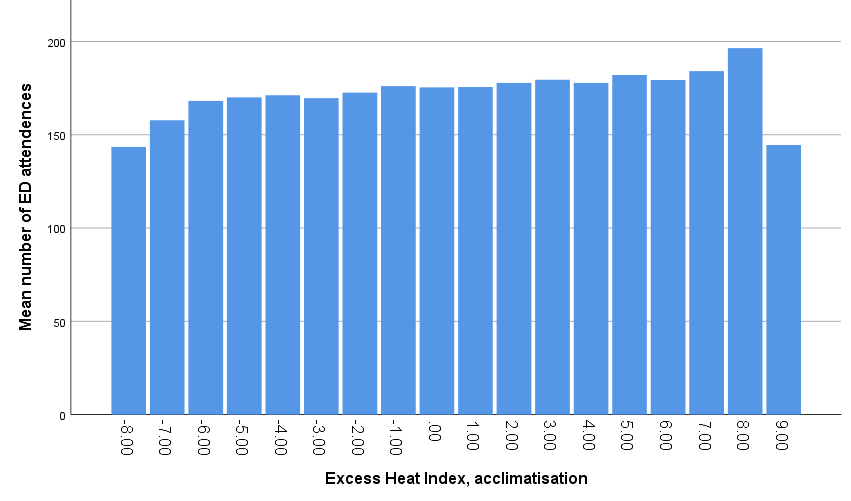 | 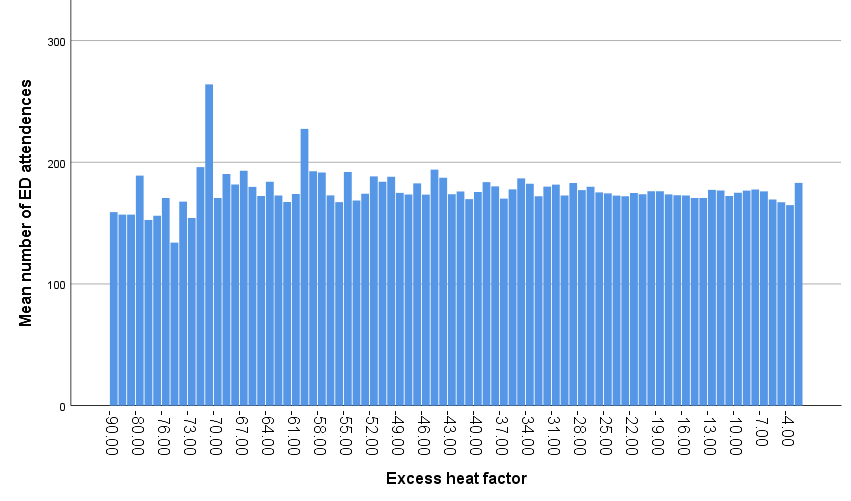 |
| **Botswana** | 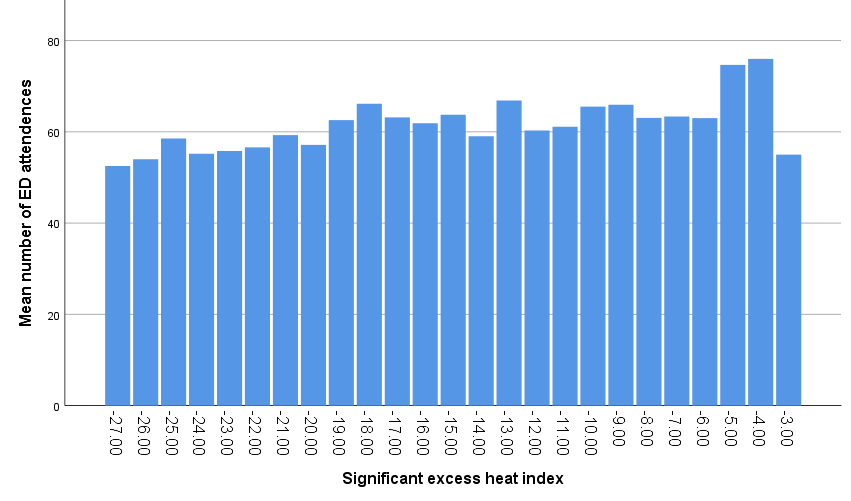 | 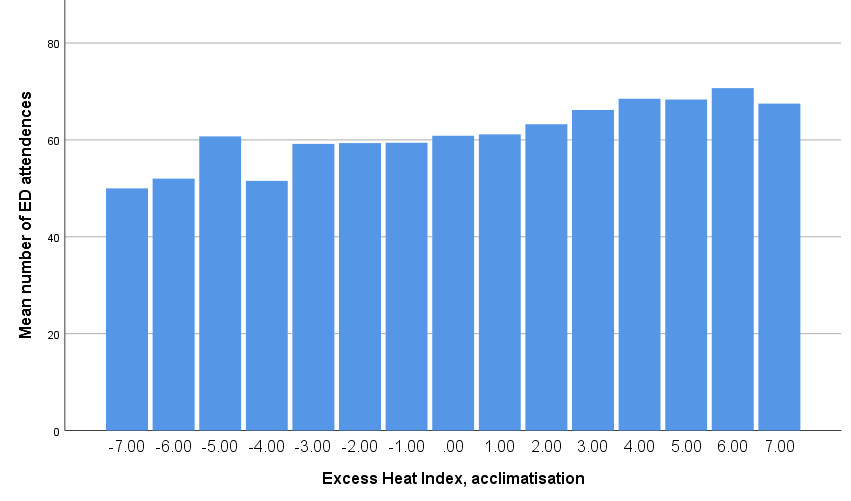 | 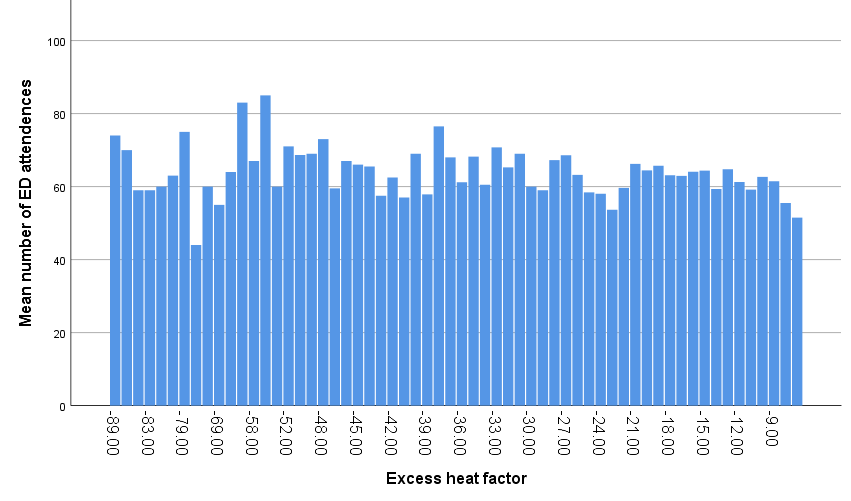 |
| **Netherlands** | 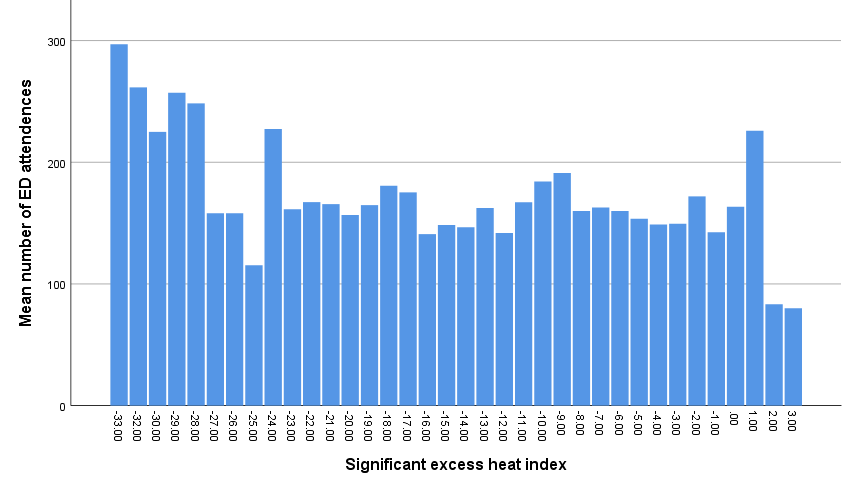 | 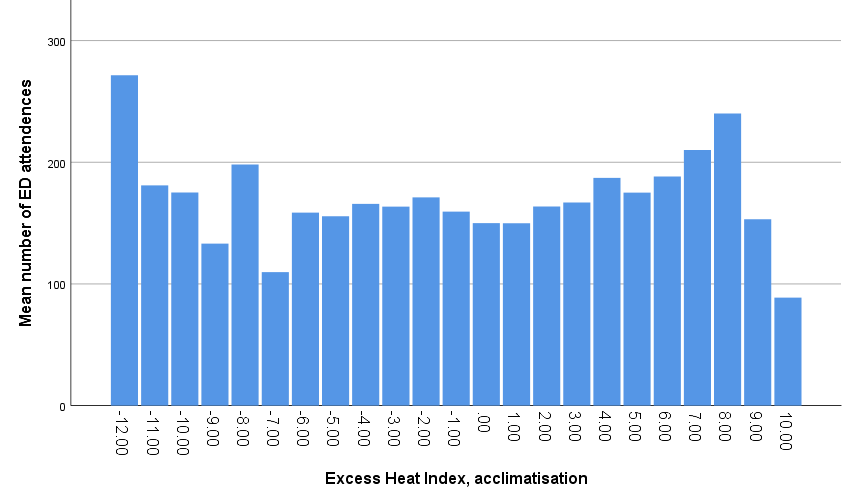 | 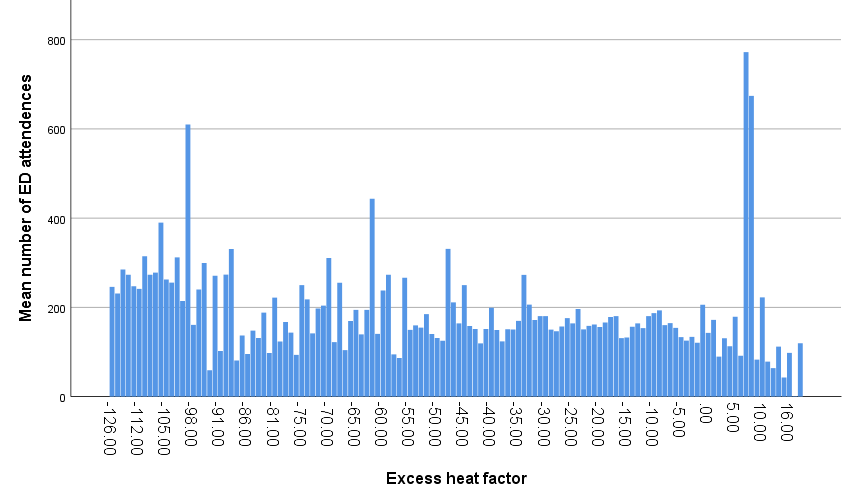 |
| **Pakistan** | 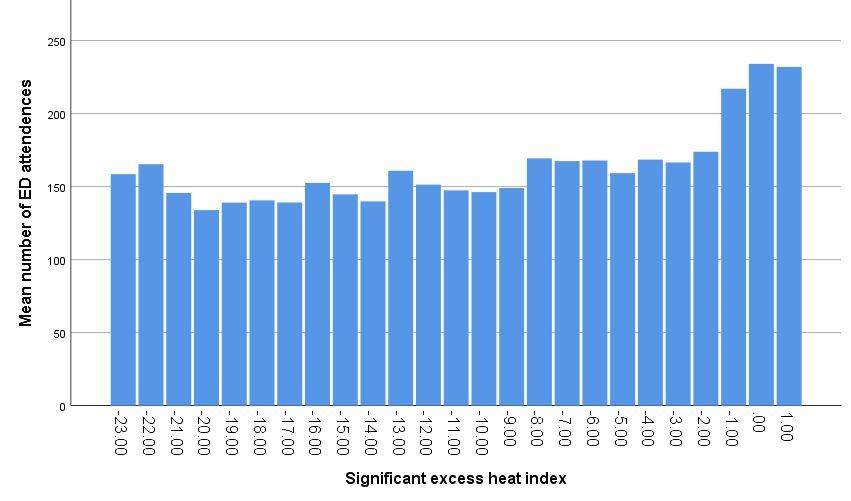 | 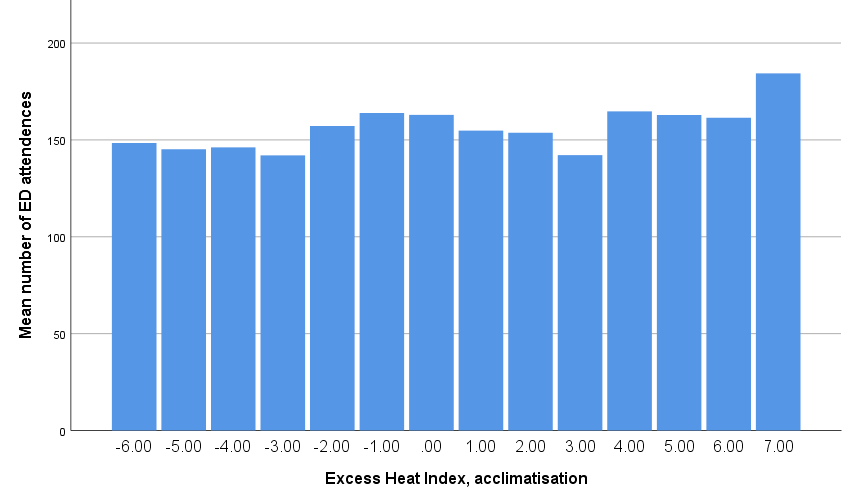 | 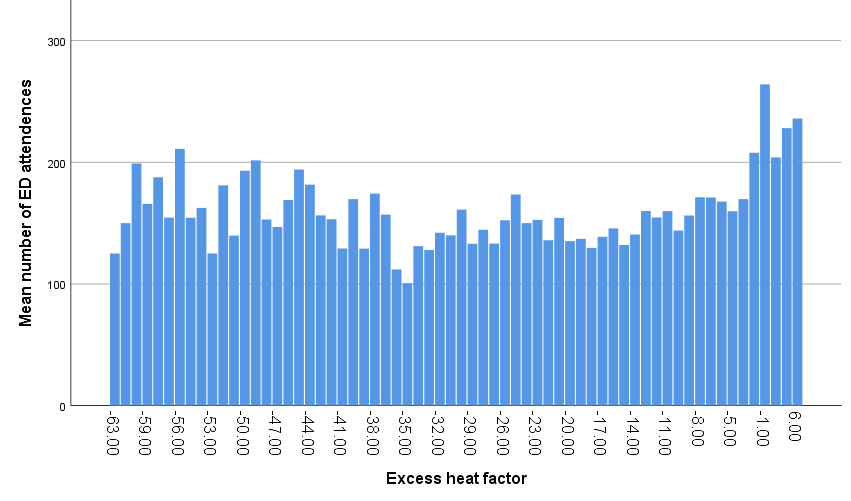 |
| **USA** | 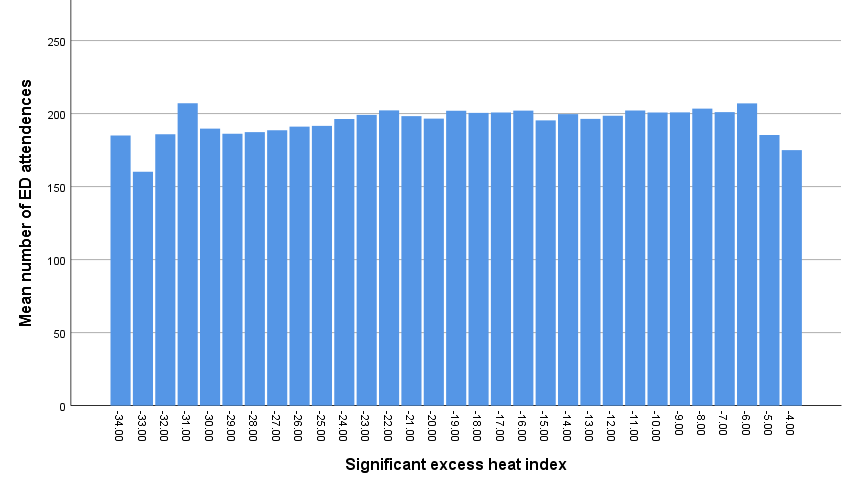 | 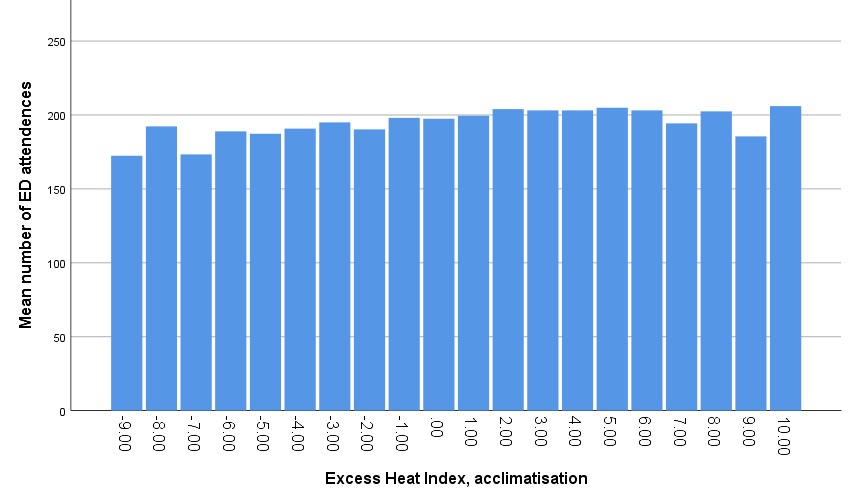 | 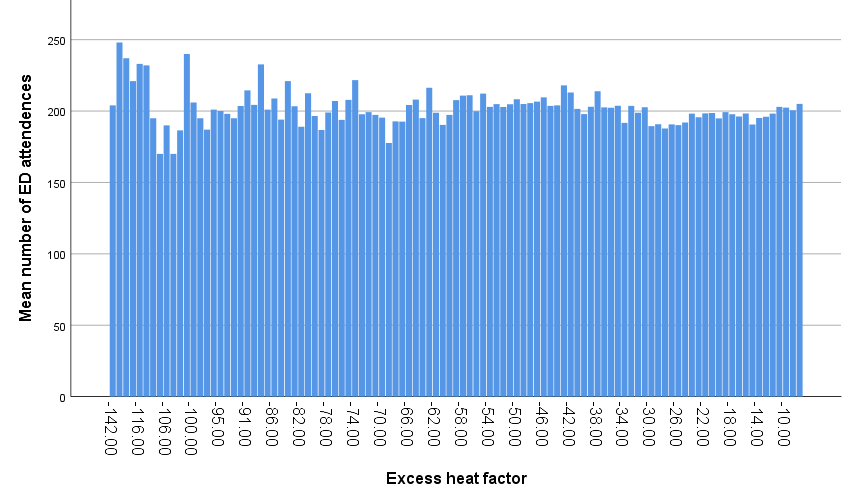 |
